# Supplementary material for: DNA methylation age from peripheral blood predicts progression to Alzheimer’s disease, white matter disease burden, and cortical atrophy
Source: NPJ Dement. 2025 May 27;1(1):7. doi: 10.1038/s44400-025-00007-1 (PMC12116384; doi:10.1038/s44400-025-00007-1)
Supplement: Supplementary file 1 — Supplementary information [file 44400_2025_7_MOESM1_ESM.docx]

**Table S1**

|  |  | Epigenetic Age with Chronologic Age | | Epigenetic Age Alone | |
| --- | --- | --- | --- | --- | --- |
|  |  | β ± SE | p-value | β ± SE | p-value |
| DNAmPhenoAge | CDR-SB | 8.41E-3 ± 2.72E-3 | 1.95E-03 | 0.01 ± 1.84E-3 | 3.81E-11 |
|  | MocA | -0.01 ± 4.64E-3 | 5.01E-03 | -0.01 ± 3.27E-3 | 1.93E-04 |
|  | MMSE | -0.02 ± 4.36E-3 | 9.25E-06 | -0.02 ± 3.04E-3 | 3.26E-08 |
| DNAmGrimAge | CDR-SB | 7.68E-4 ± 5.06E-3 | 0.88 | 0.01 ± 2.71E-3 | 1.68E-07 |
|  | MocA | 5.35E-3 ± 9.24E-3 | 0.56 | -8.54E-3 ± 4.74E-3 | 0.07 |
|  | MMSE | 1.33E-3 ± 8.95E-3 | 0.88 | -0.01 ± 4.52E-3 | 4.66E-03 |

**Table S1 Legend**: Summary statistics are shown for longitudinal analyses of cognitive and clinical tests in the combined control and mild cognitive impairment cohort. Advanced epigenetic age as measured by DNAmPhenoAge associated with worsened clinical scores as measured by CDR-SB, MoCA, and MMSE even after covarying for chronologic age. Linear mixed-effects analyses were performed covarying for chronologic age, sex, education, baseline score, and *APOE* ε4 dose. β, Beta coefficient from regression analysis; SE, Standard Error; CDR-SB, Clinical Dementia Rating scale Sum of Boxes; CN, cognitively normal; MoCA, Montreal Cognitive Assessment.

**Table S2**

|  | CN | MCI | AD |  |
| --- | --- | --- | --- | --- |
|  | 102 | 195 | 38 | p-value |
| Age at baseline (years; mean (SD)) | 75.0 (6.3) | 72.1 (7.1) | 74.6 (8.0) | 0.00 |
| Sex (Male (%)) | 44 (43.1%) | 114 (58.5%) | 18 (47%%) | 0.03 |
| Education (years; mean (SD)) | 16.5 (2.7) | 16.4 (2.6) | 16.1 (2.7%) | 0.49 |
| CDRSB (mean (SD)) | 0.1 (0.3) | 1.4 (1.1) | 4.9 (2.2) | <0.001 |
| MOCA (mean (SD)) | 26.0 (2.4) | 23.9 (3.3) | 19.0 (4.8) | <0.001 |
| *APOE* ε4 dosage (Count (%)) |  |  |  | <0.001 |
| 0 | 77 (75.5%) | 109 (55.9%) | 6 (15.8%) |  |
| 1 | 23 (22.5%) | 70 (35.9%) | 26 (68.4%) |  |
| 2 | 2 (2.0%) | 16 (8.2%) | 6 (15.8%) |  |

**Table S2 Legend**: Summary statistics are shown for study participants with FreeSurfer data summarized by diagnostic category with two-tailed p-values from ANOVA (continuous traits) or chi-square (categorical values) shown. CDR-SB, Clinical Dementia Rating scale Sum of Boxes; CN, cognitively normal; MoCA, Montreal Cognitive Assessment; MCI, mild cognitive impairment; AD, Alzheimer’s disease.

**Table S3**

| Laterality | Region | Beta | Standard Error | T-Value | Raw p-value | FDR p-value |
| --- | --- | --- | --- | --- | --- | --- |
| **Left** | Superior temporal | -7.80E-03 | 1.14E-03 | -6.83 | 4.02E-11 | 1.37E-09 |
|  | Lingual | -4.20E-03 | 7.91E-04 | -5.31 | 2.06E-07 | 2.80E-06 |
|  | Medial orbitofrontal | -4.79E-03 | 9.20E-04 | -5.21 | 3.40E-07 | 3.30E-06 |
|  | Superior frontal | -4.73E-03 | 9.13E-04 | -5.18 | 3.91E-07 | 3.32E-06 |
|  | Precuneus | -4.24E-03 | 8.48E-04 | -5.00 | 9.22E-07 | 5.22E-06 |
|  | Middle temporal | -5.60E-03 | 1.15E-03 | -4.85 | 1.88E-06 | 9.83E-06 |
|  | Entorhinal | -1.29E-02 | 2.72E-03 | -4.75 | 3.06E-06 | 1.39E-05 |
|  | Lateral orbitofrontal | -4.34E-03 | 9.24E-04 | -4.69 | 3.98E-06 | 1.69E-05 |
|  | Precentral | -4.46E-03 | 1.02E-03 | -4.38 | 1.62E-05 | 6.48E-05 |
|  | Lateral occipital | -3.93E-03 | 9.34E-04 | -4.20 | 3.42E-05 | 1.22E-04 |
|  | Postcentral | -3.37E-03 | 8.22E-04 | -4.10 | 5.15E-05 | 1.59E-04 |
|  | Pars triangularis | -3.89E-03 | 9.76E-04 | -3.98 | 8.39E-05 | 2.38E-04 |
|  | Supramarginal | -3.80E-03 | 9.69E-04 | -3.93 | 1.04E-04 | 2.84E-04 |
|  | Rostral middle frontal | -2.95E-03 | 7.62E-04 | -3.87 | 1.33E-04 | 3.35E-04 |
|  | Temporal pole | -8.60E-03 | 2.28E-03 | -3.76 | 1.98E-04 | 4.47E-04 |
|  | Inferior temporal | -4.37E-03 | 1.17E-03 | -3.73 | 2.25E-04 | 4.78E-04 |
|  | Pars orbitalis | -4.55E-03 | 1.26E-03 | -3.61 | 3.55E-04 | 6.52E-04 |
|  | Isthmus cingulate | -4.56E-03 | 1.27E-03 | -3.60 | 3.68E-04 | 6.59E-04 |
|  | Transverse temporal | -4.83E-03 | 1.37E-03 | -3.53 | 4.71E-04 | 8.12E-04 |
|  | Superior parietal | -3.03E-03 | 8.74E-04 | -3.47 | 5.97E-04 | 9.90E-04 |
|  | Parahippocampal | -7.53E-03 | 2.19E-03 | -3.45 | 6.42E-04 | 1.04E-03 |
|  | Fusiform | -3.75E-03 | 1.14E-03 | -3.30 | 1.08E-03 | 1.67E-03 |
|  | Pars opercularis | -2.73E-03 | 8.35E-04 | -3.27 | 1.17E-03 | 1.77E-03 |
|  | Pericalcarine | -2.58E-03 | 7.93E-04 | -3.25 | 1.26E-03 | 1.86E-03 |
|  | Caudal middle frontal | -2.99E-03 | 9.45E-04 | -3.17 | 1.69E-03 | 2.44E-03 |
|  | Cuneus | -2.62E-03 | 8.30E-04 | -3.15 | 1.77E-03 | 2.51E-03 |
|  | Inferior parietal | -2.98E-03 | 9.59E-04 | -3.11 | 2.06E-03 | 2.86E-03 |
|  | Paracentral | -2.58E-03 | 9.61E-04 | -2.69 | 0.01 | 0.01 |
|  | Insula | -2.64E-03 | 1.12E-03 | -2.35 | 0.02 | 0.02 |
|  | Banks of the superior temporal sulcus | -2.78E-03 | 1.19E-03 | -2.33 | 0.02 | 0.02 |
|  | Frontalpole | -3.31E-03 | 1.64E-03 | -2.02 | 0.04 | 0.05 |
|  | Posterior Cingulate | -1.31E-03 | 9.84E-04 | -1.33 | 0.18 | 0.20 |
|  | Caudal anterior cingulate | 2.40E-03 | 1.96E-03 | 1.23 | 0.22 | 0.24 |
|  | Rostral anterior cingulate | -3.54E-05 | 1.60E-03 | -0.02 | 0.98 | 0.98 |
| **Right** | Superior temporal | -7.45E-03 | 9.77E-04 | -7.63 | 2.59E-13 | 1.76E-11 |
|  | Precentral | -5.53E-03 | 9.63E-04 | -5.74 | 2.15E-08 | 4.10E-07 |
|  | Lingual | -4.77E-03 | 8.34E-04 | -5.72 | 2.41E-08 | 4.10E-07 |
|  | Supramarginal | -4.60E-03 | 8.84E-04 | -5.21 | 3.36E-07 | 3.30E-06 |
|  | Middle temporal | -5.43E-03 | 1.06E-03 | -5.13 | 4.87E-07 | 3.68E-06 |
|  | Lateral occipital | -5.08E-03 | 9.95E-04 | -5.10 | 5.65E-07 | 3.84E-06 |
|  | Precuneus | -4.32E-03 | 8.57E-04 | -5.04 | 7.83E-07 | 4.84E-06 |
|  | Inferior temporal | -5.43E-03 | 1.14E-03 | -4.76 | 2.95E-06 | 1.39E-05 |
|  | Fusiform | -4.91E-03 | 1.12E-03 | -4.36 | 1.72E-05 | 6.50E-05 |
|  | Pars triangularis | -3.74E-03 | 9.07E-04 | -4.12 | 4.81E-05 | 1.59E-04 |
|  | Superior frontal | -3.50E-03 | 8.50E-04 | -4.11 | 4.95E-05 | 1.59E-04 |
|  | Entorhinal | -1.20E-02 | 2.99E-03 | -3.99 | 8.02E-05 | 2.37E-04 |
|  | Temporal pole | -9.96E-03 | 2.54E-03 | -3.92 | 1.10E-04 | 2.87E-04 |
|  | Inferior parietal | -3.75E-03 | 9.86E-04 | -3.80 | 1.74E-04 | 4.22E-04 |
|  | Transverse temporal | -5.53E-03 | 1.47E-03 | -3.77 | 1.91E-04 | 4.47E-04 |
|  | Parahippocampal | -6.76E-03 | 1.80E-03 | -3.76 | 2.04E-04 | 4.47E-04 |
|  | Isthmus cingulate | -4.81E-03 | 1.30E-03 | -3.70 | 2.57E-04 | 5.29E-04 |
|  | Banks of the superior temporal sulcus | -3.74E-03 | 1.02E-03 | -3.68 | 2.70E-04 | 5.40E-04 |
|  | Pars orbitalis | -4.56E-03 | 1.24E-03 | -3.67 | 2.78E-04 | 5.40E-04 |
|  | Postcentral | -2.99E-03 | 8.16E-04 | -3.66 | 2.95E-04 | 5.57E-04 |
|  | Superior parietal | -3.27E-03 | 9.27E-04 | -3.53 | 4.78E-04 | 8.12E-04 |
|  | Caudal middle frontal | -3.13E-03 | 9.14E-04 | -3.42 | 6.93E-04 | 1.10E-03 |
|  | Pericalcarine | -2.35E-03 | 7.69E-04 | -3.06 | 2.42E-03 | 3.30E-03 |
|  | Cuneus | -2.53E-03 | 8.78E-04 | -2.88 | 4.22E-03 | 0.01 |
|  | Pars opercularis | -2.43E-03 | 8.59E-04 | -2.82 | 0.01 | 0.01 |
|  | Insula | -3.04E-03 | 1.14E-03 | -2.66 | 0.01 | 0.01 |
|  | Paracentral | -2.46E-03 | 1.00E-03 | -2.46 | 0.01 | 0.02 |
|  | Lateral orbitofrontal | -2.33E-03 | 9.72E-04 | -2.40 | 0.02 | 0.02 |
|  | Rostral middle frontal | -1.59E-03 | 7.51E-04 | -2.12 | 0.04 | 0.04 |
|  | Caudal anterior cingulate | 2.46E-03 | 1.74E-03 | 1.41 | 0.16 | 0.18 |
|  | Frontalpole | -1.91E-03 | 1.64E-03 | -1.17 | 0.24 | 0.26 |
|  | Medial orbitofrontal | -1.13E-03 | 1.08E-03 | -1.04 | 0.30 | 0.31 |
|  | Rostral anterior cingulate | 1.53E-03 | 1.62E-03 | 0.95 | 0.34 | 0.36 |
|  | Posterior Cingulate | 9.31E-04 | 1.05E-03 | 0.89 | 0.38 | 0.38 |

Table S3 Legend: Associations between DNAmPhenoAge and cortical thickness are shown across the spectrum of normal aging to neurodegenerative disease. All analyses were performed using multiple regression covarying for sex, education, CDR-SB score, and APOE ε4 dose.

**Table S4**

| Laterality | Region | Beta | Standard Error | T-value | Raw p-value | FDR p-value |
| --- | --- | --- | --- | --- | --- | --- |
| **Left** | Superior temporal | -1.21E-02 | 1.63E-03 | -7.39 | 1.27E-12 | 4.32E-11 |
|  | Middle temporal | -9.64E-03 | 1.64E-03 | -5.88 | 1.03E-08 | 1.89E-07 |
|  | Superior frontal | -7.60E-03 | 1.31E-03 | -5.82 | 1.39E-08 | 1.89E-07 |
|  | Precentral | -7.42E-03 | 1.46E-03 | -5.09 | 5.93E-07 | 3.40E-06 |
|  | Lateral orbitofrontal | -6.72E-03 | 1.33E-03 | -5.06 | 7.02E-07 | 3.67E-06 |
|  | Isthmus cingulate | -8.50E-03 | 1.81E-03 | -4.71 | 3.70E-06 | 1.58E-05 |
|  | Medial orbitofrontal | -6.29E-03 | 1.34E-03 | -4.69 | 3.95E-06 | 1.58E-05 |
|  | Entorhinal | -1.83E-02 | 3.93E-03 | -4.65 | 4.74E-06 | 1.79E-05 |
|  | Postcentral | -5.41E-03 | 1.18E-03 | -4.58 | 6.56E-06 | 2.35E-05 |
|  | Rostral middle frontal | -4.96E-03 | 1.09E-03 | -4.55 | 7.66E-06 | 2.60E-05 |
|  | Precuneus | -5.57E-03 | 1.23E-03 | -4.52 | 8.74E-06 | 2.83E-05 |
|  | Supramarginal | -6.16E-03 | 1.39E-03 | -4.43 | 1.27E-05 | 3.93E-05 |
|  | Lingual | -5.12E-03 | 1.16E-03 | -4.42 | 1.33E-05 | 3.93E-05 |
|  | Lateral occipital | -5.91E-03 | 1.35E-03 | -4.39 | 1.53E-05 | 4.34E-05 |
|  | Fusiform | -6.95E-03 | 1.63E-03 | -4.28 | 2.51E-05 | 6.37E-05 |
|  | Pars triangularis | -5.79E-03 | 1.41E-03 | -4.11 | 4.91E-05 | 1.11E-04 |
|  | Inferior temporal | -6.63E-03 | 1.69E-03 | -3.93 | 1.04E-04 | 2.08E-04 |
|  | Caudal middle frontal | -5.23E-03 | 1.36E-03 | -3.86 | 1.35E-04 | 2.56E-04 |
|  | Temporal pole | -1.27E-02 | 3.30E-03 | -3.86 | 1.35E-04 | 2.56E-04 |
|  | Superior parietal | -4.68E-03 | 1.26E-03 | -3.72 | 2.35E-04 | 4.22E-04 |
|  | Pars opercularis | -4.46E-03 | 1.20E-03 | -3.72 | 2.36E-04 | 4.22E-04 |
|  | Transverse temporal | -7.32E-03 | 1.97E-03 | -3.71 | 2.46E-04 | 4.28E-04 |
|  | Inferior parietal | -4.47E-03 | 1.38E-03 | -3.23 | 1.36E-03 | 2.20E-03 |
|  | Pars orbitalis | -5.64E-03 | 1.83E-03 | -3.08 | 2.23E-03 | 3.44E-03 |
|  | Banks of the superior temporal sulcus | -5.11E-03 | 1.71E-03 | -2.98 | 3.05E-03 | 4.51E-03 |
|  | Parahippocampal | -8.35E-03 | 3.18E-03 | -2.63 | 0.01 | 0.01 |
|  | Insula | -4.24E-03 | 1.62E-03 | -2.62 | 0.01 | 0.01 |
|  | Paracentral | -3.53E-03 | 1.39E-03 | -2.54 | 0.01 | 0.02 |
|  | Frontalpole | -5.89E-03 | 2.37E-03 | -2.49 | 0.01 | 0.02 |
|  | Pericalcarine | -2.38E-03 | 1.16E-03 | -2.06 | 0.04 | 0.05 |
|  | Cuneus | -2.35E-03 | 1.21E-03 | -1.94 | 0.05 | 0.06 |
|  | Caudal anterior cingulate | 4.31E-03 | 2.82E-03 | 1.53 | 0.13 | 0.14 |
|  | Posterior Cingulate | -1.98E-03 | 1.42E-03 | -1.39 | 0.16 | 0.18 |
|  | Rostral anterior cingulate | 1.71E-03 | 2.30E-03 | 0.74 | 0.46 | 0.47 |
| **Right** | Superior temporal | -1.10E-02 | 1.41E-03 | -7.82 | 7.11E-14 | 4.83E-12 |
|  | Fusiform | -9.30E-03 | 1.59E-03 | -5.85 | 1.20E-08 | 1.89E-07 |
|  | Precentral | -8.03E-03 | 1.39E-03 | -5.78 | 1.73E-08 | 1.96E-07 |
|  | Middle temporal | -8.66E-03 | 1.51E-03 | -5.73 | 2.33E-08 | 2.26E-07 |
|  | Inferior temporal | -8.85E-03 | 1.63E-03 | -5.42 | 1.16E-07 | 9.86E-07 |
|  | Supramarginal | -6.62E-03 | 1.28E-03 | -5.19 | 3.76E-07 | 2.84E-06 |
|  | Lateral occipital | -7.32E-03 | 1.44E-03 | -5.09 | 5.91E-07 | 3.40E-06 |
|  | Superior frontal | -6.17E-03 | 1.21E-03 | -5.09 | 6.00E-07 | 3.40E-06 |
|  | Lingual | -5.83E-03 | 1.22E-03 | -4.77 | 2.73E-06 | 1.33E-05 |
|  | Inferior parietal | -6.61E-03 | 1.41E-03 | -4.69 | 3.93E-06 | 1.58E-05 |
|  | Precuneus | -5.46E-03 | 1.25E-03 | -4.37 | 1.65E-05 | 4.49E-05 |
|  | Isthmus cingulate | -7.98E-03 | 1.87E-03 | -4.27 | 2.53E-05 | 6.37E-05 |
|  | Banks of the superior temporal sulcus | -6.21E-03 | 1.46E-03 | -4.26 | 2.67E-05 | 6.48E-05 |
|  | Pars orbitalis | -7.43E-03 | 1.78E-03 | -4.16 | 4.04E-05 | 9.47E-05 |
|  | Entorhinal | -1.75E-02 | 4.32E-03 | -4.06 | 6.25E-05 | 1.37E-04 |
|  | Pars triangularis | -5.19E-03 | 1.31E-03 | -3.96 | 9.29E-05 | 1.97E-04 |
|  | Temporal pole | -1.45E-02 | 3.67E-03 | -3.94 | 9.96E-05 | 2.05E-04 |
|  | Postcentral | -4.36E-03 | 1.18E-03 | -3.70 | 2.54E-04 | 4.32E-04 |
|  | Superior parietal | -4.78E-03 | 1.34E-03 | -3.57 | 4.09E-04 | 6.78E-04 |
|  | Caudal middle frontal | -4.26E-03 | 1.32E-03 | -3.22 | 1.40E-03 | 2.22E-03 |
|  | Pars opercularis | -3.81E-03 | 1.24E-03 | -3.07 | 2.28E-03 | 3.45E-03 |
|  | Parahippocampal | -7.76E-03 | 2.62E-03 | -2.96 | 3.27E-03 | 4.73E-03 |
|  | Insula | -4.75E-03 | 1.65E-03 | -2.88 | 4.23E-03 | 0.01 |
|  | Lateral orbitofrontal | -3.96E-03 | 1.40E-03 | -2.83 | 4.93E-03 | 0.01 |
|  | Transverse temporal | -5.32E-03 | 2.14E-03 | -2.48 | 0.01 | 0.02 |
|  | Rostral middle frontal | -2.52E-03 | 1.08E-03 | -2.33 | 0.02 | 0.03 |
|  | Paracentral | -3.05E-03 | 1.45E-03 | -2.10 | 0.04 | 0.04 |
|  | Cuneus | -2.65E-03 | 1.28E-03 | -2.08 | 0.04 | 0.05 |
|  | Pericalcarine | -2.27E-03 | 1.12E-03 | -2.03 | 0.04 | 0.05 |
|  | Frontal pole | -3.85E-03 | 2.36E-03 | -1.63 | 0.10 | 0.12 |
|  | Caudal anterior cingulate | 3.40E-03 | 2.51E-03 | 1.35 | 0.18 | 0.19 |
|  | Medial orbitofrontal | -1.48E-03 | 1.56E-03 | -0.95 | 0.34 | 0.36 |
|  | Rostral anterior cingulate | 1.49E-03 | 2.34E-03 | 0.64 | 0.52 | 0.53 |
|  | Posterior cingulate | 3.23E-04 | 1.52E-03 | 0.21 | 0.83 | 0.83 |

**Table S4 Legend**: Associations between DNAmGrimAge and cortical thickness are shown across the spectrum of normal aging to neurodegenerative disease. All analyses were performed using multiple regression covarying for sex, education, CDR-SB score, and APOE ε4 dose.

**Table S5**

| **Diagnosis** | **Epigenetic Score** | **Laterality** | **Region** | **Beta** | **Standard Error** | **T-value** | **Raw p-value** | **FDR p-value** |
| --- | --- | --- | --- | --- | --- | --- | --- | --- |
| AD | DNAmPhenoAge | Left | Inferior parietal | 6.45E-03 | 3.00E-03 | 2.15 | 0.04 | 0.84 |
| AD | DNAmPhenoAge | Left | Frontalpole | 9.72E-03 | 4.88E-03 | 1.99 | 0.06 | 0.84 |
| AD | DNAmPhenoAge | Left | Parahippocampal | -1.36E-02 | 7.11E-03 | -1.92 | 0.06 | 0.84 |
| AD | DNAmPhenoAge | Left | Caudal anterior cingulate | 1.21E-02 | 6.41E-03 | 1.88 | 0.07 | 0.84 |
| AD | DNAmPhenoAge | Left | Medial orbitofrontal | -5.44E-03 | 2.97E-03 | -1.83 | 0.08 | 0.84 |
| AD | DNAmPhenoAge | Left | Isthmus cingulate | -7.21E-03 | 4.12E-03 | -1.75 | 0.09 | 0.84 |
| AD | DNAmPhenoAge | Left | Pars triangularis | -4.77E-03 | 3.47E-03 | -1.37 | 0.18 | 0.84 |
| AD | DNAmPhenoAge | Left | Entorhinal | -1.27E-02 | 9.54E-03 | -1.33 | 0.19 | 0.84 |
| AD | DNAmPhenoAge | Left | Lateral orbitofrontal | -4.03E-03 | 3.09E-03 | -1.31 | 0.20 | 0.84 |
| AD | DNAmPhenoAge | Left | Caudal middle frontal | 4.20E-03 | 3.27E-03 | 1.28 | 0.21 | 0.84 |
| AD | DNAmPhenoAge | Left | Insula | -4.77E-03 | 3.86E-03 | -1.23 | 0.23 | 0.84 |
| AD | DNAmPhenoAge | Left | Rostral anterior cingulate | -6.79E-03 | 5.68E-03 | -1.19 | 0.24 | 0.84 |
| AD | DNAmPhenoAge | Left | Superior parietal | 3.02E-03 | 2.77E-03 | 1.09 | 0.28 | 0.84 |
| AD | DNAmPhenoAge | Left | Superior temporal | -3.44E-03 | 3.42E-03 | -1.01 | 0.32 | 0.84 |
| AD | DNAmPhenoAge | Left | Pars orbitalis | -3.76E-03 | 4.04E-03 | -0.93 | 0.36 | 0.84 |
| AD | DNAmPhenoAge | Left | Lingual | -1.54E-03 | 1.73E-03 | -0.89 | 0.38 | 0.84 |
| AD | DNAmPhenoAge | Left | Pars opercularis | -2.46E-03 | 2.84E-03 | -0.87 | 0.39 | 0.84 |
| AD | DNAmPhenoAge | Left | Posterior Cingulate | 2.43E-03 | 2.89E-03 | 0.84 | 0.41 | 0.84 |
| AD | DNAmPhenoAge | Left | Supramarginal | 2.17E-03 | 2.64E-03 | 0.82 | 0.42 | 0.84 |
| AD | DNAmPhenoAge | Left | Precuneus | 1.70E-03 | 2.40E-03 | 0.71 | 0.48 | 0.84 |
| AD | DNAmPhenoAge | Left | Temporal pole | -6.53E-03 | 9.75E-03 | -0.67 | 0.51 | 0.84 |
| AD | DNAmPhenoAge | Left | Cuneus | 1.49E-03 | 2.38E-03 | 0.63 | 0.54 | 0.84 |
| AD | DNAmPhenoAge | Left | Transverse temporal | 2.14E-03 | 3.60E-03 | 0.60 | 0.56 | 0.84 |
| AD | DNAmPhenoAge | Left | Paracentral | 1.98E-03 | 3.46E-03 | 0.57 | 0.57 | 0.84 |
| AD | DNAmPhenoAge | Left | Banks of the superior temporal sulcus | -1.77E-03 | 3.29E-03 | -0.54 | 0.59 | 0.84 |
| AD | DNAmPhenoAge | Left | Precentral | -1.49E-03 | 3.12E-03 | -0.48 | 0.64 | 0.86 |
| AD | DNAmPhenoAge | Left | Pericalcarine | -1.16E-03 | 2.50E-03 | -0.46 | 0.65 | 0.86 |
| AD | DNAmPhenoAge | Left | Rostral middle frontal | 8.67E-04 | 2.46E-03 | 0.35 | 0.73 | 0.90 |
| AD | DNAmPhenoAge | Left | Inferior temporal | -1.18E-03 | 3.91E-03 | -0.30 | 0.77 | 0.90 |
| AD | DNAmPhenoAge | Left | Postcentral | -6.07E-04 | 2.42E-03 | -0.25 | 0.80 | 0.90 |
| AD | DNAmPhenoAge | Left | Middle temporal | -9.96E-04 | 4.02E-03 | -0.25 | 0.81 | 0.90 |
| AD | DNAmPhenoAge | Left | Lateral occipital | 6.05E-04 | 2.85E-03 | 0.21 | 0.83 | 0.91 |
| AD | DNAmPhenoAge | Left | Fusiform | -3.91E-04 | 4.05E-03 | -0.10 | 0.92 | 0.97 |
| AD | DNAmPhenoAge | Left | Superior frontal | 1.02E-04 | 3.26E-03 | 0.03 | 0.98 | 0.98 |
| AD | DNAmPhenoAge | Right | Pars orbitalis | -7.27E-03 | 3.57E-03 | -2.04 | 0.05 | 0.84 |
| AD | DNAmPhenoAge | Right | Cuneus | 3.83E-03 | 2.53E-03 | 1.51 | 0.14 | 0.84 |
| AD | DNAmPhenoAge | Right | Inferior parietal | 4.40E-03 | 2.99E-03 | 1.47 | 0.15 | 0.84 |
| AD | DNAmPhenoAge | Right | Posterior Cingulate | 4.08E-03 | 3.19E-03 | 1.28 | 0.21 | 0.84 |
| AD | DNAmPhenoAge | Right | Precentral | -3.97E-03 | 3.13E-03 | -1.27 | 0.21 | 0.84 |
| AD | DNAmPhenoAge | Right | Pars triangularis | -3.68E-03 | 3.23E-03 | -1.14 | 0.26 | 0.84 |
| AD | DNAmPhenoAge | Right | Precuneus | 2.75E-03 | 2.56E-03 | 1.07 | 0.29 | 0.84 |
| AD | DNAmPhenoAge | Right | Pericalcarine | 2.10E-03 | 2.16E-03 | 0.97 | 0.34 | 0.84 |
| AD | DNAmPhenoAge | Right | Parahippocampal | -5.19E-03 | 5.65E-03 | -0.92 | 0.37 | 0.84 |
| AD | DNAmPhenoAge | Right | Temporal pole | -1.01E-02 | 1.14E-02 | -0.89 | 0.38 | 0.84 |
| AD | DNAmPhenoAge | Right | Entorhinal | -1.03E-02 | 1.20E-02 | -0.86 | 0.40 | 0.84 |
| AD | DNAmPhenoAge | Right | Superior temporal | -2.70E-03 | 3.24E-03 | -0.83 | 0.41 | 0.84 |
| AD | DNAmPhenoAge | Right | Medial orbitofrontal | -2.82E-03 | 3.42E-03 | -0.82 | 0.42 | 0.84 |
| AD | DNAmPhenoAge | Right | Lateral occipital | 2.25E-03 | 2.85E-03 | 0.79 | 0.44 | 0.84 |
| AD | DNAmPhenoAge | Right | Superior parietal | 2.06E-03 | 2.64E-03 | 0.78 | 0.44 | 0.84 |
| AD | DNAmPhenoAge | Right | Inferior temporal | -3.66E-03 | 4.79E-03 | -0.76 | 0.45 | 0.84 |
| AD | DNAmPhenoAge | Right | Paracentral | 2.21E-03 | 3.10E-03 | 0.71 | 0.48 | 0.84 |
| AD | DNAmPhenoAge | Right | Lateral orbitofrontal | -2.28E-03 | 3.23E-03 | -0.71 | 0.49 | 0.84 |
| AD | DNAmPhenoAge | Right | Insula | -3.01E-03 | 4.48E-03 | -0.67 | 0.51 | 0.84 |
| AD | DNAmPhenoAge | Right | Caudal middle frontal | 2.05E-03 | 3.23E-03 | 0.63 | 0.53 | 0.84 |
| AD | DNAmPhenoAge | Right | Rostral middle frontal | 1.58E-03 | 2.61E-03 | 0.61 | 0.55 | 0.84 |
| AD | DNAmPhenoAge | Right | Isthmus cingulate | -1.96E-03 | 3.34E-03 | -0.59 | 0.56 | 0.84 |
| AD | DNAmPhenoAge | Right | Supramarginal | 1.38E-03 | 2.47E-03 | 0.56 | 0.58 | 0.84 |
| AD | DNAmPhenoAge | Right | Postcentral | 1.07E-03 | 2.53E-03 | 0.42 | 0.68 | 0.86 |
| AD | DNAmPhenoAge | Right | Pars opercularis | -1.04E-03 | 2.45E-03 | -0.42 | 0.68 | 0.86 |
| AD | DNAmPhenoAge | Right | Transverse temporal | 1.69E-03 | 4.06E-03 | 0.42 | 0.68 | 0.86 |
| AD | DNAmPhenoAge | Right | Rostral anterior cingulate | 1.87E-03 | 4.52E-03 | 0.41 | 0.68 | 0.86 |
| AD | DNAmPhenoAge | Right | Lingual | -6.80E-04 | 2.14E-03 | -0.32 | 0.75 | 0.90 |
| AD | DNAmPhenoAge | Right | Banks of the superior temporal sulcus | 9.25E-04 | 3.08E-03 | 0.30 | 0.77 | 0.90 |
| AD | DNAmPhenoAge | Right | Fusiform | 1.16E-03 | 4.22E-03 | 0.27 | 0.79 | 0.90 |
| AD | DNAmPhenoAge | Right | Caudal anterior cingulate | -5.30E-04 | 5.02E-03 | -0.11 | 0.92 | 0.97 |
| AD | DNAmPhenoAge | Right | Frontalpole | 4.57E-04 | 5.59E-03 | 0.08 | 0.94 | 0.97 |
| AD | DNAmPhenoAge | Right | Middle temporal | -3.55E-04 | 4.44E-03 | -0.08 | 0.94 | 0.97 |
| AD | DNAmPhenoAge | Right | Superior frontal | -1.55E-04 | 3.02E-03 | -0.05 | 0.96 | 0.97 |
| CN | DNAmPhenoAge | Left | Superior parietal | -7.15E-03 | 1.40E-03 | -5.10 | 1.72E-06 | 7.79E-05 |
| CN | DNAmPhenoAge | Left | Superior temporal | -8.69E-03 | 1.92E-03 | -4.54 | 1.67E-05 | 2.84E-04 |
| CN | DNAmPhenoAge | Left | Lateral occipital | -6.42E-03 | 1.49E-03 | -4.29 | 4.23E-05 | 3.60E-04 |
| CN | DNAmPhenoAge | Left | Precuneus | -5.95E-03 | 1.44E-03 | -4.12 | 8.05E-05 | 5.47E-04 |
| CN | DNAmPhenoAge | Left | Superior frontal | -6.43E-03 | 1.57E-03 | -4.09 | 8.88E-05 | 5.49E-04 |
| CN | DNAmPhenoAge | Left | Precentral | -7.42E-03 | 1.84E-03 | -4.04 | 1.08E-04 | 5.65E-04 |
| CN | DNAmPhenoAge | Left | Middle temporal | -6.85E-03 | 1.78E-03 | -3.85 | 2.13E-04 | 9.66E-04 |
| CN | DNAmPhenoAge | Left | Postcentral | -5.67E-03 | 1.52E-03 | -3.73 | 3.29E-04 | 1.20E-03 |
| CN | DNAmPhenoAge | Left | Supramarginal | -5.94E-03 | 1.60E-03 | -3.72 | 3.35E-04 | 1.20E-03 |
| CN | DNAmPhenoAge | Left | Rostral middle frontal | -5.35E-03 | 1.46E-03 | -3.68 | 3.94E-04 | 1.34E-03 |
| CN | DNAmPhenoAge | Left | Lingual | -5.29E-03 | 1.45E-03 | -3.65 | 4.36E-04 | 1.41E-03 |
| CN | DNAmPhenoAge | Left | Paracentral | -6.42E-03 | 1.77E-03 | -3.63 | 4.58E-04 | 1.42E-03 |
| CN | DNAmPhenoAge | Left | Caudal middle frontal | -4.91E-03 | 1.56E-03 | -3.14 | 2.22E-03 | 0.01 |
| CN | DNAmPhenoAge | Left | Cuneus | -4.82E-03 | 1.63E-03 | -2.96 | 3.86E-03 | 0.01 |
| CN | DNAmPhenoAge | Left | Inferior parietal | -5.08E-03 | 1.73E-03 | -2.95 | 4.05E-03 | 0.01 |
| CN | DNAmPhenoAge | Left | Medial orbitofrontal | -5.00E-03 | 1.84E-03 | -2.72 | 0.01 | 0.02 |
| CN | DNAmPhenoAge | Left | Lateral orbitofrontal | -4.74E-03 | 1.75E-03 | -2.72 | 0.01 | 0.02 |
| CN | DNAmPhenoAge | Left | Fusiform | -4.21E-03 | 1.57E-03 | -2.68 | 0.01 | 0.02 |
| CN | DNAmPhenoAge | Left | Pars triangularis | -4.92E-03 | 2.06E-03 | -2.39 | 0.02 | 0.03 |
| CN | DNAmPhenoAge | Left | Frontalpole | -6.92E-03 | 2.93E-03 | -2.36 | 0.02 | 0.03 |
| CN | DNAmPhenoAge | Left | Pericalcarine | -3.35E-03 | 1.53E-03 | -2.19 | 0.03 | 0.05 |
| CN | DNAmPhenoAge | Left | Transverse temporal | -5.22E-03 | 2.50E-03 | -2.09 | 0.04 | 0.06 |
| CN | DNAmPhenoAge | Left | Entorhinal | -6.84E-03 | 3.58E-03 | -1.91 | 0.06 | 0.08 |
| CN | DNAmPhenoAge | Left | Posterior Cingulate | -3.56E-03 | 1.90E-03 | -1.87 | 0.06 | 0.08 |
| CN | DNAmPhenoAge | Left | Pars opercularis | -2.90E-03 | 1.60E-03 | -1.81 | 0.07 | 0.10 |
| CN | DNAmPhenoAge | Left | Inferior temporal | -2.98E-03 | 1.76E-03 | -1.69 | 0.09 | 0.12 |
| CN | DNAmPhenoAge | Left | Isthmus cingulate | -2.41E-03 | 2.14E-03 | -1.12 | 0.26 | 0.31 |
| CN | DNAmPhenoAge | Left | Pars orbitalis | -2.72E-03 | 2.53E-03 | -1.08 | 0.28 | 0.33 |
| CN | DNAmPhenoAge | Left | Temporal pole | -3.00E-03 | 3.14E-03 | -0.95 | 0.34 | 0.39 |
| CN | DNAmPhenoAge | Left | Insula | -1.72E-03 | 1.88E-03 | -0.91 | 0.36 | 0.41 |
| CN | DNAmPhenoAge | Left | Rostral anterior cingulate | 2.66E-03 | 2.98E-03 | 0.89 | 0.37 | 0.41 |
| CN | DNAmPhenoAge | Left | Banks of the superior temporal sulcus | -1.67E-03 | 2.13E-03 | -0.79 | 0.43 | 0.46 |
| CN | DNAmPhenoAge | Left | Parahippocampal | -1.46E-03 | 3.74E-03 | -0.39 | 0.70 | 0.70 |
| CN | DNAmPhenoAge | Left | Caudal anterior cingulate | 1.44E-03 | 3.77E-03 | 0.38 | 0.70 | 0.70 |
| CN | DNAmPhenoAge | Right | Superior temporal | -7.91E-03 | 1.57E-03 | -5.03 | 2.29E-06 | 7.79E-05 |
| CN | DNAmPhenoAge | Right | Superior parietal | -7.44E-03 | 1.51E-03 | -4.93 | 3.47E-06 | 7.87E-05 |
| CN | DNAmPhenoAge | Right | Supramarginal | -7.16E-03 | 1.60E-03 | -4.47 | 2.20E-05 | 2.99E-04 |
| CN | DNAmPhenoAge | Right | Lateral occipital | -6.91E-03 | 1.59E-03 | -4.36 | 3.35E-05 | 3.56E-04 |
| CN | DNAmPhenoAge | Right | Precentral | -7.49E-03 | 1.73E-03 | -4.33 | 3.66E-05 | 3.56E-04 |
| CN | DNAmPhenoAge | Right | Precuneus | -6.50E-03 | 1.56E-03 | -4.18 | 6.59E-05 | 4.98E-04 |
| CN | DNAmPhenoAge | Right | Inferior parietal | -6.91E-03 | 1.71E-03 | -4.04 | 1.08E-04 | 5.65E-04 |
| CN | DNAmPhenoAge | Right | Caudal middle frontal | -5.96E-03 | 1.51E-03 | -3.96 | 1.46E-04 | 7.09E-04 |
| CN | DNAmPhenoAge | Right | Lingual | -5.42E-03 | 1.43E-03 | -3.80 | 2.57E-04 | 1.09E-03 |
| CN | DNAmPhenoAge | Right | Postcentral | -5.73E-03 | 1.54E-03 | -3.72 | 3.35E-04 | 1.20E-03 |
| CN | DNAmPhenoAge | Right | Inferior temporal | -6.76E-03 | 1.92E-03 | -3.51 | 6.87E-04 | 2.03E-03 |
| CN | DNAmPhenoAge | Right | Paracentral | -6.77E-03 | 1.94E-03 | -3.49 | 7.44E-04 | 2.11E-03 |
| CN | DNAmPhenoAge | Right | Transverse temporal | -9.28E-03 | 2.72E-03 | -3.42 | 9.37E-04 | 2.55E-03 |
| CN | DNAmPhenoAge | Right | Superior frontal | -4.86E-03 | 1.45E-03 | -3.35 | 1.17E-03 | 3.06E-03 |
| CN | DNAmPhenoAge | Right | Rostral middle frontal | -4.65E-03 | 1.44E-03 | -3.22 | 1.74E-03 | 4.38E-03 |
| CN | DNAmPhenoAge | Right | Pericalcarine | -4.39E-03 | 1.41E-03 | -3.11 | 2.45E-03 | 0.01 |
| CN | DNAmPhenoAge | Right | Middle temporal | -5.44E-03 | 1.76E-03 | -3.09 | 2.65E-03 | 0.01 |
| CN | DNAmPhenoAge | Right | Pars triangularis | -4.80E-03 | 1.72E-03 | -2.80 | 0.01 | 0.02 |
| CN | DNAmPhenoAge | Right | Fusiform | -4.48E-03 | 1.65E-03 | -2.71 | 0.01 | 0.02 |
| CN | DNAmPhenoAge | Right | Cuneus | -4.07E-03 | 1.68E-03 | -2.42 | 0.02 | 0.03 |
| CN | DNAmPhenoAge | Right | Banks of the superior temporal sulcus | -4.13E-03 | 1.86E-03 | -2.22 | 0.03 | 0.05 |
| CN | DNAmPhenoAge | Right | Temporal pole | -7.81E-03 | 3.70E-03 | -2.11 | 0.04 | 0.06 |
| CN | DNAmPhenoAge | Right | Frontalpole | -5.89E-03 | 2.91E-03 | -2.02 | 0.05 | 0.07 |
| CN | DNAmPhenoAge | Right | Lateral orbitofrontal | -3.70E-03 | 1.86E-03 | -1.99 | 0.05 | 0.07 |
| CN | DNAmPhenoAge | Right | Entorhinal | -8.08E-03 | 4.29E-03 | -1.88 | 0.06 | 0.08 |
| CN | DNAmPhenoAge | Right | Pars opercularis | -2.78E-03 | 1.63E-03 | -1.71 | 0.09 | 0.12 |
| CN | DNAmPhenoAge | Right | Medial orbitofrontal | -3.16E-03 | 2.08E-03 | -1.52 | 0.13 | 0.17 |
| CN | DNAmPhenoAge | Right | Isthmus cingulate | -3.62E-03 | 2.41E-03 | -1.50 | 0.14 | 0.18 |
| CN | DNAmPhenoAge | Right | Pars orbitalis | -3.56E-03 | 2.43E-03 | -1.46 | 0.15 | 0.19 |
| CN | DNAmPhenoAge | Right | Parahippocampal | -3.58E-03 | 2.83E-03 | -1.27 | 0.21 | 0.26 |
| CN | DNAmPhenoAge | Right | Insula | -1.80E-03 | 2.03E-03 | -0.89 | 0.38 | 0.42 |
| CN | DNAmPhenoAge | Right | Posterior Cingulate | 1.60E-03 | 1.99E-03 | 0.81 | 0.42 | 0.45 |
| CN | DNAmPhenoAge | Right | Caudal anterior cingulate | 2.65E-03 | 3.67E-03 | 0.72 | 0.47 | 0.49 |
| CN | DNAmPhenoAge | Right | Rostral anterior cingulate | 1.74E-03 | 3.41E-03 | 0.51 | 0.61 | 0.63 |
| MCI | DNAmPhenoAge | Left | Superior temporal | -7.80E-03 | 1.59E-03 | -4.90 | 2.07E-06 | 7.04E-05 |
| MCI | DNAmPhenoAge | Left | Entorhinal | -1.58E-02 | 3.77E-03 | -4.20 | 4.17E-05 | 5.67E-04 |
| MCI | DNAmPhenoAge | Left | Medial orbitofrontal | -4.66E-03 | 1.14E-03 | -4.09 | 6.52E-05 | 7.39E-04 |
| MCI | DNAmPhenoAge | Left | Temporal pole | -1.19E-02 | 3.02E-03 | -3.93 | 1.18E-04 | 9.75E-04 |
| MCI | DNAmPhenoAge | Left | Precuneus | -4.44E-03 | 1.14E-03 | -3.91 | 1.29E-04 | 9.75E-04 |
| MCI | DNAmPhenoAge | Left | Lingual | -4.00E-03 | 1.11E-03 | -3.60 | 4.11E-04 | 2.00E-03 |
| MCI | DNAmPhenoAge | Left | Superior frontal | -4.23E-03 | 1.20E-03 | -3.51 | 5.54E-04 | 2.46E-03 |
| MCI | DNAmPhenoAge | Left | Middle temporal | -5.47E-03 | 1.58E-03 | -3.46 | 6.59E-04 | 2.52E-03 |
| MCI | DNAmPhenoAge | Left | Pars orbitalis | -5.39E-03 | 1.58E-03 | -3.40 | 8.22E-04 | 2.79E-03 |
| MCI | DNAmPhenoAge | Left | Lateral orbitofrontal | -3.98E-03 | 1.21E-03 | -3.28 | 1.23E-03 | 3.70E-03 |
| MCI | DNAmPhenoAge | Left | Inferior temporal | -5.37E-03 | 1.64E-03 | -3.28 | 1.25E-03 | 3.70E-03 |
| MCI | DNAmPhenoAge | Left | Parahippocampal | -9.28E-03 | 2.97E-03 | -3.13 | 2.05E-03 | 0.01 |
| MCI | DNAmPhenoAge | Left | Isthmus cingulate | -5.10E-03 | 1.67E-03 | -3.05 | 2.63E-03 | 0.01 |
| MCI | DNAmPhenoAge | Left | Transverse temporal | -5.54E-03 | 1.85E-03 | -3.00 | 3.09E-03 | 0.01 |
| MCI | DNAmPhenoAge | Left | Inferior parietal | -3.70E-03 | 1.25E-03 | -2.97 | 3.36E-03 | 0.01 |
| MCI | DNAmPhenoAge | Left | Pars triangularis | -3.18E-03 | 1.19E-03 | -2.67 | 0.01 | 0.02 |
| MCI | DNAmPhenoAge | Left | Supramarginal | -3.53E-03 | 1.34E-03 | -2.64 | 0.01 | 0.02 |
| MCI | DNAmPhenoAge | Left | Lateral occipital | -3.41E-03 | 1.32E-03 | -2.58 | 0.01 | 0.02 |
| MCI | DNAmPhenoAge | Left | Rostral middle frontal | -2.40E-03 | 9.82E-04 | -2.45 | 0.02 | 0.03 |
| MCI | DNAmPhenoAge | Left | Pericalcarine | -2.56E-03 | 1.05E-03 | -2.45 | 0.02 | 0.03 |
| MCI | DNAmPhenoAge | Left | Postcentral | -2.56E-03 | 1.07E-03 | -2.40 | 0.02 | 0.03 |
| MCI | DNAmPhenoAge | Left | Caudal middle frontal | -3.03E-03 | 1.28E-03 | -2.37 | 0.02 | 0.03 |
| MCI | DNAmPhenoAge | Left | Fusiform | -3.81E-03 | 1.61E-03 | -2.37 | 0.02 | 0.03 |
| MCI | DNAmPhenoAge | Left | Cuneus | -2.46E-03 | 1.08E-03 | -2.27 | 0.02 | 0.03 |
| MCI | DNAmPhenoAge | Left | Pars opercularis | -2.39E-03 | 1.06E-03 | -2.25 | 0.03 | 0.04 |
| MCI | DNAmPhenoAge | Left | Precentral | -3.01E-03 | 1.35E-03 | -2.22 | 0.03 | 0.04 |
| MCI | DNAmPhenoAge | Left | Banks of the superior temporal sulcus | -2.98E-03 | 1.62E-03 | -1.84 | 0.07 | 0.09 |
| MCI | DNAmPhenoAge | Left | Frontalpole | -3.94E-03 | 2.16E-03 | -1.83 | 0.07 | 0.09 |
| MCI | DNAmPhenoAge | Left | Insula | -2.51E-03 | 1.53E-03 | -1.64 | 0.10 | 0.13 |
| MCI | DNAmPhenoAge | Left | Superior parietal | -1.89E-03 | 1.18E-03 | -1.59 | 0.11 | 0.14 |
| MCI | DNAmPhenoAge | Left | Paracentral | -1.55E-03 | 1.18E-03 | -1.31 | 0.19 | 0.23 |
| MCI | DNAmPhenoAge | Left | Posterior Cingulate | -9.73E-04 | 1.28E-03 | -0.76 | 0.45 | 0.51 |
| MCI | DNAmPhenoAge | Left | Caudal anterior cingulate | 9.48E-04 | 2.51E-03 | 0.38 | 0.71 | 0.77 |
| MCI | DNAmPhenoAge | Left | Rostral anterior cingulate | -5.62E-04 | 2.05E-03 | -0.27 | 0.78 | 0.83 |
| MCI | DNAmPhenoAge | Right | Superior temporal | -7.62E-03 | 1.32E-03 | -5.76 | 3.40E-08 | 2.31E-06 |
| MCI | DNAmPhenoAge | Right | Middle temporal | -6.19E-03 | 1.32E-03 | -4.70 | 5.09E-06 | 1.15E-04 |
| MCI | DNAmPhenoAge | Right | Lingual | -4.90E-03 | 1.16E-03 | -4.22 | 3.84E-05 | 5.67E-04 |
| MCI | DNAmPhenoAge | Right | Precuneus | -4.45E-03 | 1.10E-03 | -4.03 | 8.10E-05 | 7.87E-04 |
| MCI | DNAmPhenoAge | Right | Fusiform | -5.83E-03 | 1.57E-03 | -3.72 | 2.59E-04 | 1.69E-03 |
| MCI | DNAmPhenoAge | Right | Lateral occipital | -5.23E-03 | 1.41E-03 | -3.71 | 2.73E-04 | 1.69E-03 |
| MCI | DNAmPhenoAge | Right | Precentral | -4.70E-03 | 1.28E-03 | -3.67 | 3.21E-04 | 1.82E-03 |
| MCI | DNAmPhenoAge | Right | Isthmus cingulate | -6.45E-03 | 1.79E-03 | -3.60 | 4.03E-04 | 2.00E-03 |
| MCI | DNAmPhenoAge | Right | Inferior temporal | -5.02E-03 | 1.43E-03 | -3.50 | 5.78E-04 | 2.46E-03 |
| MCI | DNAmPhenoAge | Right | Supramarginal | -4.04E-03 | 1.17E-03 | -3.46 | 6.67E-04 | 2.52E-03 |
| MCI | DNAmPhenoAge | Right | Entorhinal | -1.39E-02 | 4.07E-03 | -3.41 | 7.82E-04 | 2.79E-03 |
| MCI | DNAmPhenoAge | Right | Temporal pole | -1.10E-02 | 3.29E-03 | -3.35 | 9.92E-04 | 3.21E-03 |
| MCI | DNAmPhenoAge | Right | Parahippocampal | -7.99E-03 | 2.53E-03 | -3.15 | 1.88E-03 | 0.01 |
| MCI | DNAmPhenoAge | Right | Banks of the superior temporal sulcus | -4.27E-03 | 1.36E-03 | -3.13 | 2.01E-03 | 0.01 |
| MCI | DNAmPhenoAge | Right | Pars orbitalis | -4.85E-03 | 1.63E-03 | -2.97 | 3.34E-03 | 0.01 |
| MCI | DNAmPhenoAge | Right | Pars triangularis | -3.26E-03 | 1.17E-03 | -2.78 | 0.01 | 0.02 |
| MCI | DNAmPhenoAge | Right | Superior frontal | -3.14E-03 | 1.13E-03 | -2.77 | 0.01 | 0.02 |
| MCI | DNAmPhenoAge | Right | Inferior parietal | -3.22E-03 | 1.31E-03 | -2.45 | 0.02 | 0.03 |
| MCI | DNAmPhenoAge | Right | Cuneus | -2.80E-03 | 1.16E-03 | -2.42 | 0.02 | 0.03 |
| MCI | DNAmPhenoAge | Right | Postcentral | -2.37E-03 | 1.04E-03 | -2.28 | 0.02 | 0.03 |
| MCI | DNAmPhenoAge | Right | Pars opercularis | -2.48E-03 | 1.14E-03 | -2.18 | 0.03 | 0.04 |
| MCI | DNAmPhenoAge | Right | Transverse temporal | -4.29E-03 | 1.98E-03 | -2.17 | 0.03 | 0.04 |
| MCI | DNAmPhenoAge | Right | Insula | -3.02E-03 | 1.45E-03 | -2.08 | 0.04 | 0.05 |
| MCI | DNAmPhenoAge | Right | Pericalcarine | -2.14E-03 | 1.05E-03 | -2.05 | 0.04 | 0.05 |
| MCI | DNAmPhenoAge | Right | Caudal middle frontal | -2.37E-03 | 1.24E-03 | -1.92 | 0.06 | 0.08 |
| MCI | DNAmPhenoAge | Right | Superior parietal | -1.96E-03 | 1.28E-03 | -1.53 | 0.13 | 0.16 |
| MCI | DNAmPhenoAge | Right | Caudal anterior cingulate | 2.79E-03 | 2.15E-03 | 1.29 | 0.20 | 0.23 |
| MCI | DNAmPhenoAge | Right | Lateral orbitofrontal | -1.36E-03 | 1.26E-03 | -1.07 | 0.28 | 0.32 |
| MCI | DNAmPhenoAge | Right | Rostral middle frontal | -6.31E-04 | 9.37E-04 | -0.67 | 0.50 | 0.56 |
| MCI | DNAmPhenoAge | Right | Paracentral | -7.84E-04 | 1.26E-03 | -0.62 | 0.53 | 0.58 |
| MCI | DNAmPhenoAge | Right | Frontalpole | -4.26E-04 | 2.15E-03 | -0.20 | 0.84 | 0.87 |
| MCI | DNAmPhenoAge | Right | Rostral anterior cingulate | 3.74E-04 | 1.96E-03 | 0.19 | 0.85 | 0.87 |
| MCI | DNAmPhenoAge | Right | Posterior Cingulate | 2.51E-04 | 1.40E-03 | 0.18 | 0.86 | 0.87 |
| MCI | DNAmPhenoAge | Right | Medial orbitofrontal | 2.13E-05 | 1.40E-03 | 0.02 | 0.99 | 0.99 |
| AD | DNAmGrimAge | Left | Caudal anterior cingulate | 2.10E-02 | 9.05E-03 | 2.32 | 0.03 | 0.57 |
| AD | DNAmGrimAge | Left | Isthmus cingulate | -1.14E-02 | 5.91E-03 | -1.93 | 0.06 | 0.57 |
| AD | DNAmGrimAge | Left | Medial orbitofrontal | -8.24E-03 | 4.28E-03 | -1.92 | 0.06 | 0.57 |
| AD | DNAmGrimAge | Left | Pars triangularis | -9.06E-03 | 4.92E-03 | -1.84 | 0.08 | 0.57 |
| AD | DNAmGrimAge | Left | Parahippocampal | -1.90E-02 | 1.03E-02 | -1.84 | 0.08 | 0.57 |
| AD | DNAmGrimAge | Left | Lateral orbitofrontal | -7.17E-03 | 4.41E-03 | -1.63 | 0.11 | 0.57 |
| AD | DNAmGrimAge | Left | Entorhinal | -2.02E-02 | 1.37E-02 | -1.47 | 0.15 | 0.57 |
| AD | DNAmGrimAge | Left | Inferior parietal | 6.34E-03 | 4.52E-03 | 1.40 | 0.17 | 0.61 |
| AD | DNAmGrimAge | Left | Pars orbitalis | -7.63E-03 | 5.78E-03 | -1.32 | 0.20 | 0.68 |
| AD | DNAmGrimAge | Left | Frontalpole | 9.42E-03 | 7.31E-03 | 1.29 | 0.21 | 0.68 |
| AD | DNAmGrimAge | Left | Superior temporal | -6.02E-03 | 4.91E-03 | -1.23 | 0.23 | 0.68 |
| AD | DNAmGrimAge | Left | Insula | -5.89E-03 | 5.63E-03 | -1.05 | 0.30 | 0.73 |
| AD | DNAmGrimAge | Left | Pars opercularis | -4.07E-03 | 4.10E-03 | -0.99 | 0.33 | 0.77 |
| AD | DNAmGrimAge | Left | Temporal pole | -1.31E-02 | 1.40E-02 | -0.93 | 0.36 | 0.79 |
| AD | DNAmGrimAge | Left | Superior parietal | 3.26E-03 | 4.05E-03 | 0.80 | 0.43 | 0.86 |
| AD | DNAmGrimAge | Left | Pericalcarine | -2.60E-03 | 3.61E-03 | -0.72 | 0.48 | 0.87 |
| AD | DNAmGrimAge | Left | Rostral anterior cingulate | -5.29E-03 | 8.37E-03 | -0.63 | 0.53 | 0.87 |
| AD | DNAmGrimAge | Left | Caudal middle frontal | 3.04E-03 | 4.84E-03 | 0.63 | 0.53 | 0.87 |
| AD | DNAmGrimAge | Left | Middle temporal | -3.50E-03 | 5.79E-03 | -0.60 | 0.55 | 0.87 |
| AD | DNAmGrimAge | Left | Fusiform | -3.45E-03 | 5.84E-03 | -0.59 | 0.56 | 0.87 |
| AD | DNAmGrimAge | Left | Cuneus | 2.00E-03 | 3.45E-03 | 0.58 | 0.57 | 0.87 |
| AD | DNAmGrimAge | Left | Lingual | -1.46E-03 | 2.53E-03 | -0.58 | 0.57 | 0.87 |
| AD | DNAmGrimAge | Left | Inferior temporal | -2.77E-03 | 5.65E-03 | -0.49 | 0.63 | 0.87 |
| AD | DNAmGrimAge | Left | Precentral | -1.92E-03 | 4.52E-03 | -0.42 | 0.67 | 0.87 |
| AD | DNAmGrimAge | Left | Posterior Cingulate | 1.77E-03 | 4.22E-03 | 0.42 | 0.68 | 0.87 |
| AD | DNAmGrimAge | Left | Precuneus | 1.34E-03 | 3.50E-03 | 0.38 | 0.70 | 0.87 |
| AD | DNAmGrimAge | Left | Banks of the superior temporal sulcus | -1.81E-03 | 4.77E-03 | -0.38 | 0.71 | 0.87 |
| AD | DNAmGrimAge | Left | Postcentral | -1.32E-03 | 3.50E-03 | -0.38 | 0.71 | 0.87 |
| AD | DNAmGrimAge | Left | Rostral middle frontal | -1.33E-03 | 3.56E-03 | -0.37 | 0.71 | 0.87 |
| AD | DNAmGrimAge | Left | Lateral occipital | 1.47E-03 | 4.13E-03 | 0.36 | 0.72 | 0.87 |
| AD | DNAmGrimAge | Left | Supramarginal | 8.46E-04 | 3.86E-03 | 0.22 | 0.83 | 0.94 |
| AD | DNAmGrimAge | Left | Superior frontal | -8.04E-04 | 4.72E-03 | -0.17 | 0.87 | 0.95 |
| AD | DNAmGrimAge | Left | Transverse temporal | -7.79E-04 | 5.24E-03 | -0.15 | 0.88 | 0.95 |
| AD | DNAmGrimAge | Left | Paracentral | 3.50E-04 | 5.04E-03 | 0.07 | 0.95 | 0.96 |
| AD | DNAmGrimAge | Right | Pars orbitalis | -1.40E-02 | 4.90E-03 | -2.85 | 0.01 | 0.57 |
| AD | DNAmGrimAge | Right | Cuneus | 7.18E-03 | 3.58E-03 | 2.01 | 0.05 | 0.57 |
| AD | DNAmGrimAge | Right | Pericalcarine | 5.94E-03 | 3.00E-03 | 1.98 | 0.06 | 0.57 |
| AD | DNAmGrimAge | Right | Inferior temporal | -1.28E-02 | 6.62E-03 | -1.94 | 0.06 | 0.57 |
| AD | DNAmGrimAge | Right | Temporal pole | -2.69E-02 | 1.59E-02 | -1.69 | 0.10 | 0.57 |
| AD | DNAmGrimAge | Right | Isthmus cingulate | -7.51E-03 | 4.67E-03 | -1.61 | 0.12 | 0.57 |
| AD | DNAmGrimAge | Right | Entorhinal | -2.62E-02 | 1.70E-02 | -1.54 | 0.13 | 0.57 |
| AD | DNAmGrimAge | Right | Medial orbitofrontal | -7.33E-03 | 4.84E-03 | -1.52 | 0.14 | 0.57 |
| AD | DNAmGrimAge | Right | Superior temporal | -6.90E-03 | 4.58E-03 | -1.51 | 0.14 | 0.57 |
| AD | DNAmGrimAge | Right | Pars triangularis | -6.94E-03 | 4.61E-03 | -1.51 | 0.14 | 0.57 |
| AD | DNAmGrimAge | Right | Lateral orbitofrontal | -6.68E-03 | 4.56E-03 | -1.46 | 0.15 | 0.57 |
| AD | DNAmGrimAge | Right | Middle temporal | -7.61E-03 | 6.28E-03 | -1.21 | 0.23 | 0.68 |
| AD | DNAmGrimAge | Right | Paracentral | 5.25E-03 | 4.42E-03 | 1.19 | 0.24 | 0.68 |
| AD | DNAmGrimAge | Right | Insula | -7.55E-03 | 6.40E-03 | -1.18 | 0.25 | 0.68 |
| AD | DNAmGrimAge | Right | Superior frontal | -4.95E-03 | 4.28E-03 | -1.16 | 0.26 | 0.68 |
| AD | DNAmGrimAge | Right | Precentral | -4.80E-03 | 4.56E-03 | -1.05 | 0.30 | 0.73 |
| AD | DNAmGrimAge | Right | Pars opercularis | -3.36E-03 | 3.51E-03 | -0.96 | 0.35 | 0.79 |
| AD | DNAmGrimAge | Right | Fusiform | -5.45E-03 | 6.04E-03 | -0.90 | 0.37 | 0.79 |
| AD | DNAmGrimAge | Right | Parahippocampal | -6.64E-03 | 8.21E-03 | -0.81 | 0.43 | 0.86 |
| AD | DNAmGrimAge | Right | Banks of the superior temporal sulcus | -3.01E-03 | 4.43E-03 | -0.68 | 0.50 | 0.87 |
| AD | DNAmGrimAge | Right | Rostral middle frontal | -1.98E-03 | 3.79E-03 | -0.52 | 0.60 | 0.87 |
| AD | DNAmGrimAge | Right | Transverse temporal | 2.65E-03 | 5.88E-03 | 0.45 | 0.65 | 0.87 |
| AD | DNAmGrimAge | Right | Caudal anterior cingulate | 3.26E-03 | 7.24E-03 | 0.45 | 0.66 | 0.87 |
| AD | DNAmGrimAge | Right | Lingual | 1.35E-03 | 3.09E-03 | 0.44 | 0.67 | 0.87 |
| AD | DNAmGrimAge | Right | Precuneus | 1.56E-03 | 3.77E-03 | 0.42 | 0.68 | 0.87 |
| AD | DNAmGrimAge | Right | Inferior parietal | 1.78E-03 | 4.47E-03 | 0.40 | 0.69 | 0.87 |
| AD | DNAmGrimAge | Right | Posterior Cingulate | -1.50E-03 | 4.73E-03 | -0.32 | 0.75 | 0.89 |
| AD | DNAmGrimAge | Right | Caudal middle frontal | -1.16E-03 | 4.71E-03 | -0.25 | 0.81 | 0.94 |
| AD | DNAmGrimAge | Right | Supramarginal | -8.18E-04 | 3.60E-03 | -0.23 | 0.82 | 0.94 |
| AD | DNAmGrimAge | Right | Frontalpole | -1.59E-03 | 8.09E-03 | -0.20 | 0.85 | 0.95 |
| AD | DNAmGrimAge | Right | Postcentral | -4.87E-04 | 3.67E-03 | -0.13 | 0.90 | 0.96 |
| AD | DNAmGrimAge | Right | Rostral anterior cingulate | 5.96E-04 | 6.56E-03 | 0.09 | 0.93 | 0.96 |
| AD | DNAmGrimAge | Right | Superior parietal | 3.31E-04 | 3.85E-03 | 0.09 | 0.93 | 0.96 |
| AD | DNAmGrimAge | Right | Lateral occipital | -1.74E-05 | 4.17E-03 | 0.00 | 1.00 | 1.00 |
| CN | DNAmGrimAge | Left | Superior parietal | -1.19E-02 | 2.08E-03 | -5.73 | 1.16E-07 | 2.72E-06 |
| CN | DNAmGrimAge | Left | Superior temporal | -1.47E-02 | 2.85E-03 | -5.15 | 1.43E-06 | 1.39E-05 |
| CN | DNAmGrimAge | Left | Lateral occipital | -1.08E-02 | 2.23E-03 | -4.83 | 5.15E-06 | 3.64E-05 |
| CN | DNAmGrimAge | Left | Superior frontal | -1.12E-02 | 2.33E-03 | -4.82 | 5.35E-06 | 3.64E-05 |
| CN | DNAmGrimAge | Left | Precentral | -1.28E-02 | 2.73E-03 | -4.68 | 9.39E-06 | 5.80E-05 |
| CN | DNAmGrimAge | Left | Precuneus | -9.66E-03 | 2.18E-03 | -4.44 | 2.42E-05 | 1.28E-04 |
| CN | DNAmGrimAge | Left | Postcentral | -9.94E-03 | 2.26E-03 | -4.39 | 2.95E-05 | 1.43E-04 |
| CN | DNAmGrimAge | Left | Rostral middle frontal | -9.33E-03 | 2.17E-03 | -4.30 | 4.20E-05 | 1.90E-04 |
| CN | DNAmGrimAge | Left | Supramarginal | -1.01E-02 | 2.39E-03 | -4.23 | 5.36E-05 | 2.28E-04 |
| CN | DNAmGrimAge | Left | Middle temporal | -1.12E-02 | 2.68E-03 | -4.19 | 6.20E-05 | 2.48E-04 |
| CN | DNAmGrimAge | Left | Lingual | -8.50E-03 | 2.20E-03 | -3.87 | 1.98E-04 | 6.73E-04 |
| CN | DNAmGrimAge | Left | Lateral orbitofrontal | -9.85E-03 | 2.57E-03 | -3.83 | 2.31E-04 | 7.48E-04 |
| CN | DNAmGrimAge | Left | Inferior parietal | -9.22E-03 | 2.58E-03 | -3.57 | 5.53E-04 | 1.50E-03 |
| CN | DNAmGrimAge | Left | Caudal middle frontal | -8.29E-03 | 2.35E-03 | -3.52 | 6.61E-04 | 1.73E-03 |
| CN | DNAmGrimAge | Left | Cuneus | -8.29E-03 | 2.45E-03 | -3.39 | 1.03E-03 | 2.50E-03 |
| CN | DNAmGrimAge | Left | Paracentral | -8.39E-03 | 2.74E-03 | -3.06 | 2.89E-03 | 0.01 |
| CN | DNAmGrimAge | Left | Pars triangularis | -9.38E-03 | 3.09E-03 | -3.04 | 3.06E-03 | 0.01 |
| CN | DNAmGrimAge | Left | Fusiform | -6.94E-03 | 2.38E-03 | -2.92 | 4.38E-03 | 0.01 |
| CN | DNAmGrimAge | Left | Medial orbitofrontal | -8.11E-03 | 2.79E-03 | -2.90 | 4.57E-03 | 0.01 |
| CN | DNAmGrimAge | Left | Frontalpole | -1.08E-02 | 4.46E-03 | -2.43 | 0.02 | 0.03 |
| CN | DNAmGrimAge | Left | Pars opercularis | -5.77E-03 | 2.41E-03 | -2.39 | 0.02 | 0.03 |
| CN | DNAmGrimAge | Left | Transverse temporal | -8.23E-03 | 3.80E-03 | -2.17 | 0.03 | 0.04 |
| CN | DNAmGrimAge | Left | Posterior Cingulate | -5.77E-03 | 2.89E-03 | -2.00 | 0.05 | 0.07 |
| CN | DNAmGrimAge | Left | Pars orbitalis | -7.39E-03 | 3.80E-03 | -1.94 | 0.06 | 0.08 |
| CN | DNAmGrimAge | Left | Isthmus cingulate | -6.24E-03 | 3.23E-03 | -1.93 | 0.06 | 0.08 |
| CN | DNAmGrimAge | Left | Banks of the superior temporal sulcus | -6.01E-03 | 3.20E-03 | -1.88 | 0.06 | 0.08 |
| CN | DNAmGrimAge | Left | Inferior temporal | -5.00E-03 | 2.68E-03 | -1.87 | 0.07 | 0.09 |
| CN | DNAmGrimAge | Left | Pericalcarine | -4.05E-03 | 2.35E-03 | -1.72 | 0.09 | 0.11 |
| CN | DNAmGrimAge | Left | Temporal pole | -6.63E-03 | 4.77E-03 | -1.39 | 0.17 | 0.21 |
| CN | DNAmGrimAge | Left | Entorhinal | -7.05E-03 | 5.52E-03 | -1.28 | 0.20 | 0.23 |
| CN | DNAmGrimAge | Left | Insula | -2.78E-03 | 2.87E-03 | -0.97 | 0.34 | 0.37 |
| CN | DNAmGrimAge | Left | Caudal anterior cingulate | 5.51E-03 | 5.73E-03 | 0.96 | 0.34 | 0.37 |
| CN | DNAmGrimAge | Left | Rostral anterior cingulate | 2.25E-03 | 4.56E-03 | 0.49 | 0.62 | 0.66 |
| CN | DNAmGrimAge | Left | Parahippocampal | 5.98E-04 | 5.71E-03 | 0.10 | 0.92 | 0.94 |
| CN | DNAmGrimAge | Right | Superior temporal | -1.42E-02 | 2.27E-03 | -6.25 | 1.15E-08 | 7.82E-07 |
| CN | DNAmGrimAge | Right | Lateral occipital | -1.31E-02 | 2.29E-03 | -5.73 | 1.20E-07 | 2.72E-06 |
| CN | DNAmGrimAge | Right | Superior parietal | -1.22E-02 | 2.26E-03 | -5.38 | 5.25E-07 | 8.93E-06 |
| CN | DNAmGrimAge | Right | Inferior temporal | -1.44E-02 | 2.75E-03 | -5.25 | 9.20E-07 | 1.25E-05 |
| CN | DNAmGrimAge | Right | Inferior parietal | -1.29E-02 | 2.50E-03 | -5.15 | 1.40E-06 | 1.39E-05 |
| CN | DNAmGrimAge | Right | Precentral | -1.26E-02 | 2.58E-03 | -4.86 | 4.55E-06 | 3.64E-05 |
| CN | DNAmGrimAge | Right | Fusiform | -1.06E-02 | 2.38E-03 | -4.44 | 2.45E-05 | 1.28E-04 |
| CN | DNAmGrimAge | Right | Middle temporal | -1.07E-02 | 2.59E-03 | -4.14 | 7.60E-05 | 2.87E-04 |
| CN | DNAmGrimAge | Right | Precuneus | -9.48E-03 | 2.39E-03 | -3.96 | 1.44E-04 | 5.15E-04 |
| CN | DNAmGrimAge | Right | Supramarginal | -9.25E-03 | 2.52E-03 | -3.67 | 3.96E-04 | 1.22E-03 |
| CN | DNAmGrimAge | Right | Banks of the superior temporal sulcus | -9.93E-03 | 2.73E-03 | -3.63 | 4.54E-04 | 1.29E-03 |
| CN | DNAmGrimAge | Right | Postcentral | -8.55E-03 | 2.35E-03 | -3.63 | 4.56E-04 | 1.29E-03 |
| CN | DNAmGrimAge | Right | Superior frontal | -7.70E-03 | 2.20E-03 | -3.50 | 7.19E-04 | 1.81E-03 |
| CN | DNAmGrimAge | Right | Lingual | -7.31E-03 | 2.21E-03 | -3.31 | 1.34E-03 | 3.14E-03 |
| CN | DNAmGrimAge | Right | Caudal middle frontal | -7.32E-03 | 2.36E-03 | -3.10 | 2.58E-03 | 0.01 |
| CN | DNAmGrimAge | Right | Lateral orbitofrontal | -8.46E-03 | 2.76E-03 | -3.07 | 2.83E-03 | 0.01 |
| CN | DNAmGrimAge | Right | Isthmus cingulate | -1.07E-02 | 3.55E-03 | -3.02 | 3.24E-03 | 0.01 |
| CN | DNAmGrimAge | Right | Frontalpole | -1.31E-02 | 4.33E-03 | -3.02 | 3.25E-03 | 0.01 |
| CN | DNAmGrimAge | Right | Rostral middle frontal | -6.66E-03 | 2.21E-03 | -3.01 | 3.34E-03 | 0.01 |
| CN | DNAmGrimAge | Right | Pericalcarine | -6.10E-03 | 2.17E-03 | -2.81 | 0.01 | 0.02 |
| CN | DNAmGrimAge | Right | Cuneus | -7.06E-03 | 2.54E-03 | -2.78 | 0.01 | 0.02 |
| CN | DNAmGrimAge | Right | Paracentral | -8.37E-03 | 3.03E-03 | -2.76 | 0.01 | 0.02 |
| CN | DNAmGrimAge | Right | Transverse temporal | -1.12E-02 | 4.24E-03 | -2.64 | 0.01 | 0.02 |
| CN | DNAmGrimAge | Right | Pars triangularis | -6.26E-03 | 2.65E-03 | -2.37 | 0.02 | 0.03 |
| CN | DNAmGrimAge | Right | Pars opercularis | -5.59E-03 | 2.46E-03 | -2.28 | 0.03 | 0.04 |
| CN | DNAmGrimAge | Right | Temporal pole | -1.09E-02 | 5.67E-03 | -1.92 | 0.06 | 0.08 |
| CN | DNAmGrimAge | Right | Pars orbitalis | -5.59E-03 | 3.71E-03 | -1.51 | 0.14 | 0.17 |
| CN | DNAmGrimAge | Right | Medial orbitofrontal | -4.28E-03 | 3.18E-03 | -1.35 | 0.18 | 0.21 |
| CN | DNAmGrimAge | Right | Entorhinal | -8.71E-03 | 6.60E-03 | -1.32 | 0.19 | 0.22 |
| CN | DNAmGrimAge | Right | Insula | -3.74E-03 | 3.08E-03 | -1.21 | 0.23 | 0.26 |
| CN | DNAmGrimAge | Right | Posterior Cingulate | 1.51E-03 | 3.04E-03 | 0.50 | 0.62 | 0.66 |
| CN | DNAmGrimAge | Right | Rostral anterior cingulate | -2.25E-03 | 5.20E-03 | -0.43 | 0.67 | 0.70 |
| CN | DNAmGrimAge | Right | Caudal anterior cingulate | -5.24E-04 | 5.61E-03 | -0.09 | 0.93 | 0.94 |
| CN | DNAmGrimAge | Right | Parahippocampal | 3.73E-05 | 4.35E-03 | 0.01 | 0.99 | 0.99 |
| MCI | DNAmGrimAge | Left | Superior temporal | -1.19E-02 | 2.33E-03 | -5.09 | 8.69E-07 | 4.01E-05 |
| MCI | DNAmGrimAge | Left | Middle temporal | -9.82E-03 | 2.29E-03 | -4.29 | 2.89E-05 | 5.19E-04 |
| MCI | DNAmGrimAge | Left | Entorhinal | -2.37E-02 | 5.54E-03 | -4.27 | 3.05E-05 | 5.19E-04 |
| MCI | DNAmGrimAge | Left | Isthmus cingulate | -8.90E-03 | 2.44E-03 | -3.65 | 3.43E-04 | 2.86E-03 |
| MCI | DNAmGrimAge | Left | Temporal pole | -1.62E-02 | 4.46E-03 | -3.63 | 3.60E-04 | 2.86E-03 |
| MCI | DNAmGrimAge | Left | Superior frontal | -6.41E-03 | 1.77E-03 | -3.62 | 3.79E-04 | 2.86E-03 |
| MCI | DNAmGrimAge | Left | Medial orbitofrontal | -5.58E-03 | 1.71E-03 | -3.27 | 1.29E-03 | 0.01 |
| MCI | DNAmGrimAge | Left | Inferior temporal | -7.68E-03 | 2.42E-03 | -3.18 | 1.74E-03 | 0.01 |
| MCI | DNAmGrimAge | Left | Fusiform | -7.32E-03 | 2.34E-03 | -3.13 | 2.02E-03 | 0.01 |
| MCI | DNAmGrimAge | Left | Precuneus | -5.27E-03 | 1.70E-03 | -3.10 | 2.22E-03 | 0.01 |
| MCI | DNAmGrimAge | Left | Lateral orbitofrontal | -5.35E-03 | 1.79E-03 | -2.98 | 3.22E-03 | 0.01 |
| MCI | DNAmGrimAge | Left | Transverse temporal | -7.62E-03 | 2.73E-03 | -2.79 | 0.01 | 0.02 |
| MCI | DNAmGrimAge | Left | Supramarginal | -5.30E-03 | 1.97E-03 | -2.69 | 0.01 | 0.02 |
| MCI | DNAmGrimAge | Left | Lateral occipital | -5.16E-03 | 1.94E-03 | -2.66 | 0.01 | 0.02 |
| MCI | DNAmGrimAge | Left | Caudal middle frontal | -4.94E-03 | 1.88E-03 | -2.64 | 0.01 | 0.02 |
| MCI | DNAmGrimAge | Left | Precentral | -5.15E-03 | 1.99E-03 | -2.59 | 0.01 | 0.02 |
| MCI | DNAmGrimAge | Left | Rostral middle frontal | -3.72E-03 | 1.44E-03 | -2.58 | 0.01 | 0.02 |
| MCI | DNAmGrimAge | Left | Postcentral | -3.90E-03 | 1.57E-03 | -2.48 | 0.01 | 0.02 |
| MCI | DNAmGrimAge | Left | Inferior parietal | -4.54E-03 | 1.85E-03 | -2.46 | 0.01 | 0.02 |
| MCI | DNAmGrimAge | Left | Lingual | -3.99E-03 | 1.67E-03 | -2.39 | 0.02 | 0.03 |
| MCI | DNAmGrimAge | Left | Pars triangularis | -4.05E-03 | 1.76E-03 | -2.30 | 0.02 | 0.03 |
| MCI | DNAmGrimAge | Left | Parahippocampal | -9.91E-03 | 4.42E-03 | -2.24 | 0.03 | 0.05 |
| MCI | DNAmGrimAge | Left | Insula | -4.77E-03 | 2.25E-03 | -2.12 | 0.04 | 0.06 |
| MCI | DNAmGrimAge | Left | Pars opercularis | -3.28E-03 | 1.57E-03 | -2.09 | 0.04 | 0.06 |
| MCI | DNAmGrimAge | Left | Banks of the superior temporal sulcus | -4.72E-03 | 2.38E-03 | -1.98 | 0.05 | 0.08 |
| MCI | DNAmGrimAge | Left | Frontalpole | -6.07E-03 | 3.17E-03 | -1.91 | 0.06 | 0.09 |
| MCI | DNAmGrimAge | Left | Pars orbitalis | -4.24E-03 | 2.38E-03 | -1.78 | 0.08 | 0.11 |
| MCI | DNAmGrimAge | Left | Superior parietal | -2.79E-03 | 1.74E-03 | -1.60 | 0.11 | 0.15 |
| MCI | DNAmGrimAge | Left | Paracentral | -2.28E-03 | 1.74E-03 | -1.31 | 0.19 | 0.24 |
| MCI | DNAmGrimAge | Left | Pericalcarine | -1.87E-03 | 1.56E-03 | -1.20 | 0.23 | 0.28 |
| MCI | DNAmGrimAge | Left | Posterior Cingulate | -1.24E-03 | 1.89E-03 | -0.65 | 0.51 | 0.59 |
| MCI | DNAmGrimAge | Left | Cuneus | -9.02E-04 | 1.62E-03 | -0.56 | 0.58 | 0.66 |
| MCI | DNAmGrimAge | Left | Rostral anterior cingulate | 1.50E-03 | 3.01E-03 | 0.50 | 0.62 | 0.67 |
| MCI | DNAmGrimAge | Left | Caudal anterior cingulate | -4.29E-05 | 3.70E-03 | -0.01 | 0.99 | 0.99 |
| MCI | DNAmGrimAge | Right | Superior temporal | -9.96E-03 | 1.98E-03 | -5.02 | 1.18E-06 | 4.01E-05 |
| MCI | DNAmGrimAge | Right | Fusiform | -9.19E-03 | 2.29E-03 | -4.01 | 8.86E-05 | 1.02E-03 |
| MCI | DNAmGrimAge | Right | Middle temporal | -7.88E-03 | 1.97E-03 | -4.00 | 9.02E-05 | 1.02E-03 |
| MCI | DNAmGrimAge | Right | Lingual | -6.17E-03 | 1.73E-03 | -3.56 | 4.74E-04 | 3.12E-03 |
| MCI | DNAmGrimAge | Right | Pars orbitalis | -8.42E-03 | 2.38E-03 | -3.54 | 5.05E-04 | 3.12E-03 |
| MCI | DNAmGrimAge | Right | Precentral | -6.49E-03 | 1.89E-03 | -3.43 | 7.49E-04 | 4.24E-03 |
| MCI | DNAmGrimAge | Right | Supramarginal | -5.85E-03 | 1.72E-03 | -3.40 | 8.18E-04 | 4.24E-03 |
| MCI | DNAmGrimAge | Right | Entorhinal | -2.03E-02 | 6.00E-03 | -3.38 | 8.73E-04 | 4.24E-03 |
| MCI | DNAmGrimAge | Right | Superior frontal | -5.34E-03 | 1.66E-03 | -3.22 | 1.51E-03 | 0.01 |
| MCI | DNAmGrimAge | Right | Precuneus | -5.03E-03 | 1.65E-03 | -3.04 | 2.70E-03 | 0.01 |
| MCI | DNAmGrimAge | Right | Temporal pole | -1.45E-02 | 4.88E-03 | -2.98 | 3.25E-03 | 0.01 |
| MCI | DNAmGrimAge | Right | Isthmus cingulate | -7.85E-03 | 2.66E-03 | -2.94 | 3.64E-03 | 0.01 |
| MCI | DNAmGrimAge | Right | Pars triangularis | -4.83E-03 | 1.72E-03 | -2.81 | 0.01 | 0.02 |
| MCI | DNAmGrimAge | Right | Inferior temporal | -5.87E-03 | 2.14E-03 | -2.75 | 0.01 | 0.02 |
| MCI | DNAmGrimAge | Right | Parahippocampal | -1.03E-02 | 3.75E-03 | -2.74 | 0.01 | 0.02 |
| MCI | DNAmGrimAge | Right | Lateral occipital | -5.66E-03 | 2.11E-03 | -2.68 | 0.01 | 0.02 |
| MCI | DNAmGrimAge | Right | Banks of the superior temporal sulcus | -5.07E-03 | 2.03E-03 | -2.50 | 0.01 | 0.02 |
| MCI | DNAmGrimAge | Right | Inferior parietal | -4.61E-03 | 1.93E-03 | -2.38 | 0.02 | 0.03 |
| MCI | DNAmGrimAge | Right | Pars opercularis | -3.73E-03 | 1.67E-03 | -2.23 | 0.03 | 0.05 |
| MCI | DNAmGrimAge | Right | Postcentral | -3.39E-03 | 1.53E-03 | -2.21 | 0.03 | 0.05 |
| MCI | DNAmGrimAge | Right | Insula | -3.80E-03 | 2.14E-03 | -1.77 | 0.08 | 0.11 |
| MCI | DNAmGrimAge | Right | Caudal middle frontal | -2.77E-03 | 1.83E-03 | -1.51 | 0.13 | 0.18 |
| MCI | DNAmGrimAge | Right | Pericalcarine | -2.09E-03 | 1.55E-03 | -1.35 | 0.18 | 0.24 |
| MCI | DNAmGrimAge | Right | Cuneus | -2.29E-03 | 1.72E-03 | -1.33 | 0.19 | 0.24 |
| MCI | DNAmGrimAge | Right | Caudal anterior cingulate | 4.09E-03 | 3.17E-03 | 1.29 | 0.20 | 0.25 |
| MCI | DNAmGrimAge | Right | Superior parietal | -2.20E-03 | 1.89E-03 | -1.16 | 0.25 | 0.30 |
| MCI | DNAmGrimAge | Right | Transverse temporal | -2.59E-03 | 2.94E-03 | -0.88 | 0.38 | 0.45 |
| MCI | DNAmGrimAge | Right | Paracentral | -1.26E-03 | 1.85E-03 | -0.68 | 0.49 | 0.57 |
| MCI | DNAmGrimAge | Right | Lateral orbitofrontal | -9.66E-04 | 1.86E-03 | -0.52 | 0.60 | 0.67 |
| MCI | DNAmGrimAge | Right | Rostral middle frontal | -6.84E-04 | 1.38E-03 | -0.50 | 0.62 | 0.67 |
| MCI | DNAmGrimAge | Right | Posterior Cingulate | 9.40E-04 | 2.06E-03 | 0.46 | 0.65 | 0.69 |
| MCI | DNAmGrimAge | Right | Medial orbitofrontal | 5.38E-04 | 2.06E-03 | 0.26 | 0.79 | 0.83 |
| MCI | DNAmGrimAge | Right | Frontalpole | -2.74E-04 | 3.16E-03 | -0.09 | 0.93 | 0.94 |
| MCI | DNAmGrimAge | Right | Rostral anterior cingulate | 2.41E-04 | 2.88E-03 | 0.08 | 0.93 | 0.94 |

**Table S5 Legend:** Associations between DNAmPhenoAge and DNAmGrimAge with cortical thickness stratified by diagnosis are shown. All analyses were performed using multiple regression covarying for sex, education, CDR-SB score, and APOE ε4 dose.

**Table S6**

| **Diagnosis** | **Epigenetic Score** | **Laterality** | **Region** | **Beta** | **Standard Error** | **T-value** | **Raw p-value** | **FDR p-value** |
| --- | --- | --- | --- | --- | --- | --- | --- | --- |
| AD | DNAmPhenoAge | Left | Inferior parietal | 1.06E-02 | 4.64E-03 | 2.28 | 0.03 | 0.64 |
| AD | DNAmPhenoAge | Left | Paracentral | 9.70E-03 | 5.16E-03 | 1.88 | 0.07 | 0.64 |
| AD | DNAmPhenoAge | Left | Precuneus | 5.63E-03 | 3.68E-03 | 1.53 | 0.14 | 0.66 |
| AD | DNAmPhenoAge | Left | Frontalpole | 1.17E-02 | 7.70E-03 | 1.52 | 0.14 | 0.66 |
| AD | DNAmPhenoAge | Left | Transverse temporal | 7.91E-03 | 5.51E-03 | 1.43 | 0.16 | 0.66 |
| AD | DNAmPhenoAge | Left | Caudal middle frontal | 7.30E-03 | 5.12E-03 | 1.43 | 0.16 | 0.66 |
| AD | DNAmPhenoAge | Left | Supramarginal | 5.43E-03 | 4.10E-03 | 1.32 | 0.20 | 0.67 |
| AD | DNAmPhenoAge | Left | Posterior Cingulate | 5.90E-03 | 4.49E-03 | 1.32 | 0.20 | 0.67 |
| AD | DNAmPhenoAge | Left | Superior parietal | 4.99E-03 | 4.35E-03 | 1.15 | 0.26 | 0.79 |
| AD | DNAmPhenoAge | Left | Middle temporal | 6.46E-03 | 6.10E-03 | 1.06 | 0.30 | 0.79 |
| AD | DNAmPhenoAge | Left | Temporal pole | 1.49E-02 | 1.45E-02 | 1.02 | 0.32 | 0.79 |
| AD | DNAmPhenoAge | Left | Rostral middle frontal | 3.34E-03 | 3.84E-03 | 0.87 | 0.39 | 0.79 |
| AD | DNAmPhenoAge | Left | Fusiform | 5.30E-03 | 6.26E-03 | 0.85 | 0.40 | 0.79 |
| AD | DNAmPhenoAge | Left | Parahippocampal | -9.00E-03 | 1.12E-02 | -0.80 | 0.43 | 0.80 |
| AD | DNAmPhenoAge | Left | Lateral occipital | 3.09E-03 | 4.47E-03 | 0.69 | 0.49 | 0.84 |
| AD | DNAmPhenoAge | Left | Entorhinal | 9.42E-03 | 1.41E-02 | 0.67 | 0.51 | 0.84 |
| AD | DNAmPhenoAge | Left | Superior frontal | 3.38E-03 | 5.09E-03 | 0.66 | 0.51 | 0.84 |
| AD | DNAmPhenoAge | Left | Caudal anterior cingulate | 6.55E-03 | 1.01E-02 | 0.65 | 0.52 | 0.84 |
| AD | DNAmPhenoAge | Left | Pars opercularis | -2.41E-03 | 4.49E-03 | -0.54 | 0.60 | 0.89 |
| AD | DNAmPhenoAge | Left | Pars triangularis | -2.91E-03 | 5.47E-03 | -0.53 | 0.60 | 0.89 |
| AD | DNAmPhenoAge | Left | Cuneus | 1.88E-03 | 3.76E-03 | 0.50 | 0.62 | 0.89 |
| AD | DNAmPhenoAge | Left | Isthmus cingulate | 3.00E-03 | 6.05E-03 | 0.50 | 0.62 | 0.89 |
| AD | DNAmPhenoAge | Left | Insula | -2.83E-03 | 6.09E-03 | -0.47 | 0.64 | 0.89 |
| AD | DNAmPhenoAge | Left | Postcentral | 1.55E-03 | 3.79E-03 | 0.41 | 0.69 | 0.89 |
| AD | DNAmPhenoAge | Left | Inferior temporal | 2.43E-03 | 6.12E-03 | 0.40 | 0.69 | 0.89 |
| AD | DNAmPhenoAge | Left | Banks of the superior temporal sulcus | 1.73E-03 | 5.13E-03 | 0.34 | 0.74 | 0.89 |
| AD | DNAmPhenoAge | Left | Lateral orbitofrontal | -1.52E-03 | 4.84E-03 | -0.31 | 0.76 | 0.89 |
| AD | DNAmPhenoAge | Left | Rostral anterior cingulate | -2.48E-03 | 8.93E-03 | -0.28 | 0.78 | 0.89 |
| AD | DNAmPhenoAge | Left | Precentral | 9.75E-04 | 4.89E-03 | 0.20 | 0.84 | 0.93 |
| AD | DNAmPhenoAge | Left | Superior temporal | 9.67E-04 | 5.30E-03 | 0.18 | 0.86 | 0.93 |
| AD | DNAmPhenoAge | Left | Pericalcarine | -5.95E-04 | 3.96E-03 | -0.15 | 0.88 | 0.93 |
| AD | DNAmPhenoAge | Left | Pars orbitalis | 7.34E-04 | 6.30E-03 | 0.12 | 0.91 | 0.93 |
| AD | DNAmPhenoAge | Left | Medial orbitofrontal | 4.73E-04 | 4.48E-03 | 0.11 | 0.92 | 0.93 |
| AD | DNAmPhenoAge | Left | Lingual | 1.79E-04 | 2.71E-03 | 0.07 | 0.95 | 0.95 |
| AD | DNAmPhenoAge | Right | Lateral occipital | 1.08E-02 | 4.03E-03 | 2.67 | 0.01 | 0.50 |
| AD | DNAmPhenoAge | Right | Inferior parietal | 1.15E-02 | 4.43E-03 | 2.59 | 0.01 | 0.50 |
| AD | DNAmPhenoAge | Right | Posterior Cingulate | 1.01E-02 | 4.83E-03 | 2.09 | 0.04 | 0.64 |
| AD | DNAmPhenoAge | Right | Precuneus | 7.27E-03 | 3.90E-03 | 1.86 | 0.07 | 0.64 |
| AD | DNAmPhenoAge | Right | Middle temporal | 1.18E-02 | 6.39E-03 | 1.86 | 0.07 | 0.64 |
| AD | DNAmPhenoAge | Right | Fusiform | 1.14E-02 | 6.21E-03 | 1.84 | 0.08 | 0.64 |
| AD | DNAmPhenoAge | Right | Superior parietal | 6.96E-03 | 4.00E-03 | 1.74 | 0.09 | 0.66 |
| AD | DNAmPhenoAge | Right | Rostral middle frontal | 6.20E-03 | 3.98E-03 | 1.56 | 0.13 | 0.66 |
| AD | DNAmPhenoAge | Right | Banks of the superior temporal sulcus | 7.06E-03 | 4.64E-03 | 1.52 | 0.14 | 0.66 |
| AD | DNAmPhenoAge | Right | Supramarginal | 5.52E-03 | 3.78E-03 | 1.46 | 0.16 | 0.66 |
| AD | DNAmPhenoAge | Right | Caudal middle frontal | 7.07E-03 | 4.97E-03 | 1.42 | 0.17 | 0.66 |
| AD | DNAmPhenoAge | Right | Inferior temporal | 9.21E-03 | 6.93E-03 | 1.33 | 0.19 | 0.67 |
| AD | DNAmPhenoAge | Right | Isthmus cingulate | 6.02E-03 | 4.92E-03 | 1.22 | 0.23 | 0.75 |
| AD | DNAmPhenoAge | Right | Transverse temporal | 6.75E-03 | 6.30E-03 | 1.07 | 0.29 | 0.79 |
| AD | DNAmPhenoAge | Right | Superior frontal | 4.87E-03 | 4.62E-03 | 1.06 | 0.30 | 0.79 |
| AD | DNAmPhenoAge | Right | Cuneus | 3.91E-03 | 4.00E-03 | 0.98 | 0.34 | 0.79 |
| AD | DNAmPhenoAge | Right | Postcentral | 3.84E-03 | 3.94E-03 | 0.97 | 0.34 | 0.79 |
| AD | DNAmPhenoAge | Right | Caudal anterior cingulate | -7.21E-03 | 7.77E-03 | -0.93 | 0.36 | 0.79 |
| AD | DNAmPhenoAge | Right | Superior temporal | 4.40E-03 | 4.83E-03 | 0.91 | 0.37 | 0.79 |
| AD | DNAmPhenoAge | Right | Paracentral | 4.33E-03 | 4.87E-03 | 0.89 | 0.38 | 0.79 |
| AD | DNAmPhenoAge | Right | Entorhinal | 1.55E-02 | 1.80E-02 | 0.86 | 0.40 | 0.79 |
| AD | DNAmPhenoAge | Right | Temporal pole | 1.44E-02 | 1.70E-02 | 0.85 | 0.40 | 0.79 |
| AD | DNAmPhenoAge | Right | Frontalpole | 6.89E-03 | 8.70E-03 | 0.79 | 0.43 | 0.80 |
| AD | DNAmPhenoAge | Right | Medial orbitofrontal | 3.38E-03 | 5.21E-03 | 0.65 | 0.52 | 0.84 |
| AD | DNAmPhenoAge | Right | Pars orbitalis | -3.48E-03 | 5.57E-03 | -0.62 | 0.54 | 0.85 |
| AD | DNAmPhenoAge | Right | Lateral orbitofrontal | 2.33E-03 | 4.99E-03 | 0.47 | 0.64 | 0.89 |
| AD | DNAmPhenoAge | Right | Insula | 3.10E-03 | 6.94E-03 | 0.45 | 0.66 | 0.89 |
| AD | DNAmPhenoAge | Right | Pars opercularis | 1.65E-03 | 3.82E-03 | 0.43 | 0.67 | 0.89 |
| AD | DNAmPhenoAge | Right | Pars triangularis | 1.80E-03 | 4.93E-03 | 0.37 | 0.72 | 0.89 |
| AD | DNAmPhenoAge | Right | Pericalcarine | 1.15E-03 | 3.41E-03 | 0.34 | 0.74 | 0.89 |
| AD | DNAmPhenoAge | Right | Rostral anterior cingulate | 2.16E-03 | 7.14E-03 | 0.30 | 0.76 | 0.89 |
| AD | DNAmPhenoAge | Right | Precentral | -1.41E-03 | 4.90E-03 | -0.29 | 0.78 | 0.89 |
| AD | DNAmPhenoAge | Right | Parahippocampal | 1.17E-03 | 8.81E-03 | 0.13 | 0.90 | 0.93 |
| AD | DNAmPhenoAge | Right | Lingual | 4.35E-04 | 3.37E-03 | 0.13 | 0.90 | 0.93 |
| ALL | DNAmPhenoAge | Left | Medial orbitofrontal | -4.90E-03 | 1.31E-03 | -3.73 | 2.24E-04 | 0.02 |
| ALL | DNAmPhenoAge | Left | Superior temporal | -4.32E-03 | 1.60E-03 | -2.69 | 0.01 | 0.10 |
| ALL | DNAmPhenoAge | Left | Lingual | -2.96E-03 | 1.12E-03 | -2.64 | 0.01 | 0.10 |
| ALL | DNAmPhenoAge | Left | Pericalcarine | -2.81E-03 | 1.13E-03 | -2.49 | 0.01 | 0.10 |
| ALL | DNAmPhenoAge | Left | Cuneus | -2.94E-03 | 1.18E-03 | -2.48 | 0.01 | 0.10 |
| ALL | DNAmPhenoAge | Left | Pars triangularis | -3.18E-03 | 1.39E-03 | -2.29 | 0.02 | 0.12 |
| ALL | DNAmPhenoAge | Left | Precuneus | -2.71E-03 | 1.20E-03 | -2.26 | 0.02 | 0.12 |
| ALL | DNAmPhenoAge | Left | Lateral orbitofrontal | -2.80E-03 | 1.31E-03 | -2.13 | 0.03 | 0.14 |
| ALL | DNAmPhenoAge | Left | Pars orbitalis | -3.69E-03 | 1.79E-03 | -2.06 | 0.04 | 0.15 |
| ALL | DNAmPhenoAge | Left | Superior frontal | -2.57E-03 | 1.29E-03 | -1.99 | 0.05 | 0.15 |
| ALL | DNAmPhenoAge | Left | Rostral middle frontal | -2.04E-03 | 1.08E-03 | -1.88 | 0.06 | 0.18 |
| ALL | DNAmPhenoAge | Left | Precentral | -2.70E-03 | 1.44E-03 | -1.87 | 0.06 | 0.18 |
| ALL | DNAmPhenoAge | Left | Entorhinal | -7.01E-03 | 3.85E-03 | -1.82 | 0.07 | 0.19 |
| ALL | DNAmPhenoAge | Left | Parahippocampal | -5.63E-03 | 3.11E-03 | -1.81 | 0.07 | 0.19 |
| ALL | DNAmPhenoAge | Left | Superior parietal | -1.98E-03 | 1.24E-03 | -1.59 | 0.11 | 0.22 |
| ALL | DNAmPhenoAge | Left | Caudal middle frontal | -2.12E-03 | 1.34E-03 | -1.58 | 0.12 | 0.22 |
| ALL | DNAmPhenoAge | Left | Middle temporal | -2.52E-03 | 1.63E-03 | -1.55 | 0.12 | 0.22 |
| ALL | DNAmPhenoAge | Left | Transverse temporal | -2.98E-03 | 1.95E-03 | -1.53 | 0.13 | 0.22 |
| ALL | DNAmPhenoAge | Left | Postcentral | -1.76E-03 | 1.17E-03 | -1.51 | 0.13 | 0.22 |
| ALL | DNAmPhenoAge | Left | Inferior parietal | -2.06E-03 | 1.37E-03 | -1.51 | 0.13 | 0.22 |
| ALL | DNAmPhenoAge | Left | Pars opercularis | -1.68E-03 | 1.19E-03 | -1.42 | 0.16 | 0.25 |
| ALL | DNAmPhenoAge | Left | Supramarginal | -1.91E-03 | 1.37E-03 | -1.39 | 0.17 | 0.25 |
| ALL | DNAmPhenoAge | Left | Rostral anterior cingulate | -2.93E-03 | 2.26E-03 | -1.29 | 0.20 | 0.29 |
| ALL | DNAmPhenoAge | Left | Insula | -2.03E-03 | 1.60E-03 | -1.27 | 0.21 | 0.30 |
| ALL | DNAmPhenoAge | Left | Temporal pole | -3.77E-03 | 3.23E-03 | -1.17 | 0.24 | 0.35 |
| ALL | DNAmPhenoAge | Left | Paracentral | -1.49E-03 | 1.37E-03 | -1.09 | 0.28 | 0.37 |
| ALL | DNAmPhenoAge | Left | Inferior temporal | -1.73E-03 | 1.66E-03 | -1.04 | 0.30 | 0.38 |
| ALL | DNAmPhenoAge | Left | Posterior Cingulate | -1.39E-03 | 1.40E-03 | -0.99 | 0.32 | 0.41 |
| ALL | DNAmPhenoAge | Left | Lateral occipital | -1.26E-03 | 1.32E-03 | -0.96 | 0.34 | 0.42 |
| ALL | DNAmPhenoAge | Left | Banks of the superior temporal sulcus | -1.32E-03 | 1.69E-03 | -0.78 | 0.44 | 0.50 |
| ALL | DNAmPhenoAge | Left | Frontalpole | -1.47E-03 | 2.34E-03 | -0.63 | 0.53 | 0.60 |
| ALL | DNAmPhenoAge | Left | Fusiform | -8.21E-04 | 1.61E-03 | -0.51 | 0.61 | 0.67 |
| ALL | DNAmPhenoAge | Left | Isthmus cingulate | -8.33E-04 | 1.78E-03 | -0.47 | 0.64 | 0.69 |
| ALL | DNAmPhenoAge | Left | Caudal anterior cingulate | -1.75E-04 | 2.78E-03 | -0.06 | 0.95 | 0.95 |
| ALL | DNAmPhenoAge | Right | Precentral | -3.89E-03 | 1.37E-03 | -2.85 | 4.66E-03 | 0.10 |
| ALL | DNAmPhenoAge | Right | Lingual | -3.16E-03 | 1.18E-03 | -2.68 | 0.01 | 0.10 |
| ALL | DNAmPhenoAge | Right | Transverse temporal | -5.58E-03 | 2.09E-03 | -2.67 | 0.01 | 0.10 |
| ALL | DNAmPhenoAge | Right | Precuneus | -3.16E-03 | 1.22E-03 | -2.60 | 0.01 | 0.10 |
| ALL | DNAmPhenoAge | Right | Superior temporal | -3.33E-03 | 1.36E-03 | -2.45 | 0.01 | 0.10 |
| ALL | DNAmPhenoAge | Right | Supramarginal | -3.00E-03 | 1.25E-03 | -2.39 | 0.02 | 0.10 |
| ALL | DNAmPhenoAge | Right | Pars orbitalis | -4.21E-03 | 1.77E-03 | -2.38 | 0.02 | 0.10 |
| ALL | DNAmPhenoAge | Right | Parahippocampal | -5.46E-03 | 2.56E-03 | -2.13 | 0.03 | 0.14 |
| ALL | DNAmPhenoAge | Right | Pars triangularis | -2.66E-03 | 1.29E-03 | -2.06 | 0.04 | 0.15 |
| ALL | DNAmPhenoAge | Right | Caudal middle frontal | -2.62E-03 | 1.30E-03 | -2.01 | 0.05 | 0.15 |
| ALL | DNAmPhenoAge | Right | Medial orbitofrontal | -3.04E-03 | 1.53E-03 | -1.99 | 0.05 | 0.15 |
| ALL | DNAmPhenoAge | Right | Pericalcarine | -2.15E-03 | 1.10E-03 | -1.96 | 0.05 | 0.16 |
| ALL | DNAmPhenoAge | Right | Rostral middle frontal | -1.86E-03 | 1.07E-03 | -1.74 | 0.08 | 0.21 |
| ALL | DNAmPhenoAge | Right | Postcentral | -1.99E-03 | 1.16E-03 | -1.71 | 0.09 | 0.21 |
| ALL | DNAmPhenoAge | Right | Pars opercularis | -2.04E-03 | 1.22E-03 | -1.67 | 0.10 | 0.22 |
| ALL | DNAmPhenoAge | Right | Cuneus | -2.04E-03 | 1.25E-03 | -1.63 | 0.10 | 0.22 |
| ALL | DNAmPhenoAge | Right | Middle temporal | -2.36E-03 | 1.49E-03 | -1.59 | 0.11 | 0.22 |
| ALL | DNAmPhenoAge | Right | Isthmus cingulate | -2.93E-03 | 1.85E-03 | -1.59 | 0.11 | 0.22 |
| ALL | DNAmPhenoAge | Right | Paracentral | -2.24E-03 | 1.43E-03 | -1.57 | 0.12 | 0.22 |
| ALL | DNAmPhenoAge | Right | Superior frontal | -1.87E-03 | 1.21E-03 | -1.55 | 0.12 | 0.22 |
| ALL | DNAmPhenoAge | Right | Lateral orbitofrontal | -2.13E-03 | 1.39E-03 | -1.54 | 0.13 | 0.22 |
| ALL | DNAmPhenoAge | Right | Insula | -2.36E-03 | 1.63E-03 | -1.45 | 0.15 | 0.24 |
| ALL | DNAmPhenoAge | Right | Superior parietal | -1.85E-03 | 1.32E-03 | -1.41 | 0.16 | 0.25 |
| ALL | DNAmPhenoAge | Right | Lateral occipital | -1.55E-03 | 1.39E-03 | -1.12 | 0.27 | 0.37 |
| ALL | DNAmPhenoAge | Right | Temporal pole | -3.89E-03 | 3.60E-03 | -1.08 | 0.28 | 0.37 |
| ALL | DNAmPhenoAge | Right | Entorhinal | -4.41E-03 | 4.23E-03 | -1.04 | 0.30 | 0.38 |
| ALL | DNAmPhenoAge | Right | Posterior Cingulate | 1.40E-03 | 1.50E-03 | 0.93 | 0.35 | 0.43 |
| ALL | DNAmPhenoAge | Right | Banks of the superior temporal sulcus | -1.24E-03 | 1.43E-03 | -0.87 | 0.39 | 0.46 |
| ALL | DNAmPhenoAge | Right | Inferior parietal | -1.19E-03 | 1.39E-03 | -0.86 | 0.39 | 0.46 |
| ALL | DNAmPhenoAge | Right | Inferior temporal | -8.72E-04 | 1.59E-03 | -0.55 | 0.58 | 0.65 |
| ALL | DNAmPhenoAge | Right | Rostral anterior cingulate | -9.35E-04 | 2.30E-03 | -0.41 | 0.68 | 0.72 |
| ALL | DNAmPhenoAge | Right | Caudal anterior cingulate | -9.90E-04 | 2.46E-03 | -0.40 | 0.69 | 0.72 |
| ALL | DNAmPhenoAge | Right | Frontalpole | -8.03E-04 | 2.33E-03 | -0.34 | 0.73 | 0.75 |
| ALL | DNAmPhenoAge | Right | Fusiform | 9.37E-05 | 1.55E-03 | 0.06 | 0.95 | 0.95 |
| CN | DNAmPhenoAge | Left | Paracentral | -7.38E-03 | 2.29E-03 | -3.23 | 1.71E-03 | 0.03 |
| CN | DNAmPhenoAge | Left | Superior parietal | -5.33E-03 | 1.79E-03 | -2.97 | 3.75E-03 | 0.03 |
| CN | DNAmPhenoAge | Left | Caudal middle frontal | -5.23E-03 | 2.02E-03 | -2.59 | 0.01 | 0.05 |
| CN | DNAmPhenoAge | Left | Rostral middle frontal | -4.79E-03 | 1.88E-03 | -2.54 | 0.01 | 0.06 |
| CN | DNAmPhenoAge | Left | Superior frontal | -4.96E-03 | 2.02E-03 | -2.45 | 0.02 | 0.06 |
| CN | DNAmPhenoAge | Left | Precuneus | -4.52E-03 | 1.86E-03 | -2.43 | 0.02 | 0.06 |
| CN | DNAmPhenoAge | Left | Precentral | -5.67E-03 | 2.36E-03 | -2.40 | 0.02 | 0.06 |
| CN | DNAmPhenoAge | Left | Supramarginal | -4.88E-03 | 2.06E-03 | -2.37 | 0.02 | 0.06 |
| CN | DNAmPhenoAge | Left | Medial orbitofrontal | -5.62E-03 | 2.38E-03 | -2.36 | 0.02 | 0.06 |
| CN | DNAmPhenoAge | Left | Superior temporal | -5.55E-03 | 2.43E-03 | -2.28 | 0.02 | 0.07 |
| CN | DNAmPhenoAge | Left | Lateral occipital | -4.15E-03 | 1.90E-03 | -2.18 | 0.03 | 0.09 |
| CN | DNAmPhenoAge | Left | Middle temporal | -4.95E-03 | 2.28E-03 | -2.17 | 0.03 | 0.09 |
| CN | DNAmPhenoAge | Left | Postcentral | -4.20E-03 | 1.96E-03 | -2.15 | 0.03 | 0.09 |
| CN | DNAmPhenoAge | Left | Cuneus | -4.24E-03 | 2.11E-03 | -2.02 | 0.05 | 0.12 |
| CN | DNAmPhenoAge | Left | Lingual | -3.49E-03 | 1.86E-03 | -1.88 | 0.06 | 0.14 |
| CN | DNAmPhenoAge | Left | Fusiform | -3.66E-03 | 2.03E-03 | -1.80 | 0.08 | 0.15 |
| CN | DNAmPhenoAge | Left | Posterior Cingulate | -4.17E-03 | 2.46E-03 | -1.69 | 0.09 | 0.18 |
| CN | DNAmPhenoAge | Left | Inferior parietal | -3.60E-03 | 2.22E-03 | -1.62 | 0.11 | 0.20 |
| CN | DNAmPhenoAge | Left | Frontalpole | -6.07E-03 | 3.79E-03 | -1.60 | 0.11 | 0.20 |
| CN | DNAmPhenoAge | Left | Pericalcarine | -3.12E-03 | 1.98E-03 | -1.57 | 0.12 | 0.20 |
| CN | DNAmPhenoAge | Left | Entorhinal | -7.05E-03 | 4.64E-03 | -1.52 | 0.13 | 0.21 |
| CN | DNAmPhenoAge | Left | Lateral orbitofrontal | -3.31E-03 | 2.25E-03 | -1.47 | 0.15 | 0.23 |
| CN | DNAmPhenoAge | Left | Pars triangularis | -3.79E-03 | 2.66E-03 | -1.42 | 0.16 | 0.24 |
| CN | DNAmPhenoAge | Left | Transverse temporal | -3.31E-03 | 3.22E-03 | -1.03 | 0.31 | 0.41 |
| CN | DNAmPhenoAge | Left | Insula | -2.43E-03 | 2.44E-03 | -1.00 | 0.32 | 0.42 |
| CN | DNAmPhenoAge | Left | Pars opercularis | -1.91E-03 | 2.07E-03 | -0.92 | 0.36 | 0.46 |
| CN | DNAmPhenoAge | Left | Caudal anterior cingulate | -4.08E-03 | 4.81E-03 | -0.85 | 0.40 | 0.50 |
| CN | DNAmPhenoAge | Left | Inferior temporal | -1.53E-03 | 2.27E-03 | -0.67 | 0.50 | 0.61 |
| CN | DNAmPhenoAge | Left | Isthmus cingulate | -9.94E-04 | 2.77E-03 | -0.36 | 0.72 | 0.82 |
| CN | DNAmPhenoAge | Left | Parahippocampal | -1.61E-03 | 4.85E-03 | -0.33 | 0.74 | 0.82 |
| CN | DNAmPhenoAge | Left | Rostral anterior cingulate | 6.85E-04 | 3.85E-03 | 0.18 | 0.86 | 0.93 |
| CN | DNAmPhenoAge | Left | Temporal pole | -3.45E-04 | 4.05E-03 | -0.09 | 0.93 | 0.99 |
| CN | DNAmPhenoAge | Left | Banks of the superior temporal sulcus | -1.20E-04 | 2.75E-03 | -0.04 | 0.97 | 0.99 |
| CN | DNAmPhenoAge | Left | Pars orbitalis | -5.09E-05 | 3.25E-03 | -0.02 | 0.99 | 0.99 |
| CN | DNAmPhenoAge | Right | Caudal middle frontal | -7.24E-03 | 1.94E-03 | -3.73 | 3.28E-04 | 0.02 |
| CN | DNAmPhenoAge | Right | Supramarginal | -6.95E-03 | 2.08E-03 | -3.34 | 1.19E-03 | 0.03 |
| CN | DNAmPhenoAge | Right | Precuneus | -6.38E-03 | 2.02E-03 | -3.16 | 2.13E-03 | 0.03 |
| CN | DNAmPhenoAge | Right | Superior parietal | -6.08E-03 | 1.94E-03 | -3.13 | 2.36E-03 | 0.03 |
| CN | DNAmPhenoAge | Right | Paracentral | -7.85E-03 | 2.51E-03 | -3.12 | 2.37E-03 | 0.03 |
| CN | DNAmPhenoAge | Right | Rostral middle frontal | -5.49E-03 | 1.86E-03 | -2.95 | 4.06E-03 | 0.03 |
| CN | DNAmPhenoAge | Right | Precentral | -6.44E-03 | 2.23E-03 | -2.88 | 4.87E-03 | 0.04 |
| CN | DNAmPhenoAge | Right | Superior frontal | -5.27E-03 | 1.88E-03 | -2.80 | 0.01 | 0.04 |
| CN | DNAmPhenoAge | Right | Postcentral | -5.45E-03 | 1.99E-03 | -2.73 | 0.01 | 0.04 |
| CN | DNAmPhenoAge | Right | Transverse temporal | -9.59E-03 | 3.52E-03 | -2.72 | 0.01 | 0.04 |
| CN | DNAmPhenoAge | Right | Lingual | -4.92E-03 | 1.85E-03 | -2.66 | 0.01 | 0.05 |
| CN | DNAmPhenoAge | Right | Pars triangularis | -5.56E-03 | 2.22E-03 | -2.51 | 0.01 | 0.06 |
| CN | DNAmPhenoAge | Right | Pericalcarine | -4.30E-03 | 1.83E-03 | -2.35 | 0.02 | 0.06 |
| CN | DNAmPhenoAge | Right | Medial orbitofrontal | -5.29E-03 | 2.67E-03 | -1.98 | 0.05 | 0.12 |
| CN | DNAmPhenoAge | Right | Parahippocampal | -7.06E-03 | 3.62E-03 | -1.95 | 0.05 | 0.13 |
| CN | DNAmPhenoAge | Right | Superior temporal | -3.65E-03 | 1.92E-03 | -1.91 | 0.06 | 0.13 |
| CN | DNAmPhenoAge | Right | Inferior parietal | -4.13E-03 | 2.17E-03 | -1.90 | 0.06 | 0.13 |
| CN | DNAmPhenoAge | Right | Middle temporal | -4.06E-03 | 2.27E-03 | -1.79 | 0.08 | 0.15 |
| CN | DNAmPhenoAge | Right | Cuneus | -3.64E-03 | 2.18E-03 | -1.67 | 0.10 | 0.19 |
| CN | DNAmPhenoAge | Right | Pars orbitalis | -5.06E-03 | 3.15E-03 | -1.61 | 0.11 | 0.20 |
| CN | DNAmPhenoAge | Right | Entorhinal | -8.81E-03 | 5.56E-03 | -1.59 | 0.12 | 0.20 |
| CN | DNAmPhenoAge | Right | Lateral orbitofrontal | -3.29E-03 | 2.41E-03 | -1.36 | 0.18 | 0.27 |
| CN | DNAmPhenoAge | Right | Lateral occipital | -2.47E-03 | 1.93E-03 | -1.28 | 0.20 | 0.30 |
| CN | DNAmPhenoAge | Right | Pars opercularis | -2.57E-03 | 2.11E-03 | -1.22 | 0.23 | 0.33 |
| CN | DNAmPhenoAge | Right | Temporal pole | -5.71E-03 | 4.79E-03 | -1.19 | 0.24 | 0.33 |
| CN | DNAmPhenoAge | Right | Inferior temporal | -2.77E-03 | 2.41E-03 | -1.15 | 0.25 | 0.35 |
| CN | DNAmPhenoAge | Right | Frontalpole | -4.27E-03 | 3.76E-03 | -1.13 | 0.26 | 0.35 |
| CN | DNAmPhenoAge | Right | Insula | -2.12E-03 | 2.63E-03 | -0.81 | 0.42 | 0.52 |
| CN | DNAmPhenoAge | Right | Fusiform | -1.10E-03 | 2.07E-03 | -0.53 | 0.60 | 0.71 |
| CN | DNAmPhenoAge | Right | Banks of the superior temporal sulcus | -1.11E-03 | 2.37E-03 | -0.47 | 0.64 | 0.75 |
| CN | DNAmPhenoAge | Right | Isthmus cingulate | -1.13E-03 | 3.09E-03 | -0.37 | 0.71 | 0.82 |
| CN | DNAmPhenoAge | Right | Rostral anterior cingulate | -1.43E-03 | 4.39E-03 | -0.33 | 0.75 | 0.82 |
| CN | DNAmPhenoAge | Right | Caudal anterior cingulate | -1.16E-04 | 4.73E-03 | -0.02 | 0.98 | 0.99 |
| CN | DNAmPhenoAge | Right | Posterior Cingulate | 1.63E-05 | 2.56E-03 | 0.01 | 0.99 | 0.99 |
| MCI | DNAmPhenoAge | Left | Medial orbitofrontal | -5.25E-03 | 1.66E-03 | -3.15 | 1.89E-03 | 0.09 |
| MCI | DNAmPhenoAge | Left | Pars orbitalis | -7.00E-03 | 2.31E-03 | -3.04 | 2.73E-03 | 0.09 |
| MCI | DNAmPhenoAge | Left | Lingual | -3.38E-03 | 1.62E-03 | -2.09 | 0.04 | 0.33 |
| MCI | DNAmPhenoAge | Left | Pericalcarine | -3.12E-03 | 1.52E-03 | -2.04 | 0.04 | 0.33 |
| MCI | DNAmPhenoAge | Left | Cuneus | -3.13E-03 | 1.58E-03 | -1.98 | 0.05 | 0.33 |
| MCI | DNAmPhenoAge | Left | Precuneus | -3.25E-03 | 1.65E-03 | -1.96 | 0.05 | 0.33 |
| MCI | DNAmPhenoAge | Left | Superior temporal | -4.33E-03 | 2.30E-03 | -1.88 | 0.06 | 0.33 |
| MCI | DNAmPhenoAge | Left | Parahippocampal | -8.03E-03 | 4.33E-03 | -1.85 | 0.07 | 0.33 |
| MCI | DNAmPhenoAge | Left | Inferior parietal | -3.36E-03 | 1.82E-03 | -1.85 | 0.07 | 0.33 |
| MCI | DNAmPhenoAge | Left | Temporal pole | -8.05E-03 | 4.38E-03 | -1.84 | 0.07 | 0.33 |
| MCI | DNAmPhenoAge | Left | Transverse temporal | -4.67E-03 | 2.70E-03 | -1.73 | 0.08 | 0.34 |
| MCI | DNAmPhenoAge | Left | Rostral anterior cingulate | -5.10E-03 | 2.95E-03 | -1.73 | 0.09 | 0.34 |
| MCI | DNAmPhenoAge | Left | Entorhinal | -9.09E-03 | 5.45E-03 | -1.67 | 0.10 | 0.34 |
| MCI | DNAmPhenoAge | Left | Pars triangularis | -2.70E-03 | 1.74E-03 | -1.55 | 0.12 | 0.36 |
| MCI | DNAmPhenoAge | Left | Lateral orbitofrontal | -2.67E-03 | 1.76E-03 | -1.52 | 0.13 | 0.36 |
| MCI | DNAmPhenoAge | Left | Superior frontal | -2.24E-03 | 1.75E-03 | -1.29 | 0.20 | 0.44 |
| MCI | DNAmPhenoAge | Left | Pars opercularis | -1.98E-03 | 1.55E-03 | -1.27 | 0.20 | 0.44 |
| MCI | DNAmPhenoAge | Left | Middle temporal | -2.42E-03 | 2.28E-03 | -1.06 | 0.29 | 0.52 |
| MCI | DNAmPhenoAge | Left | Rostral middle frontal | -1.51E-03 | 1.43E-03 | -1.06 | 0.29 | 0.52 |
| MCI | DNAmPhenoAge | Left | Inferior temporal | -2.48E-03 | 2.37E-03 | -1.05 | 0.30 | 0.52 |
| MCI | DNAmPhenoAge | Left | Caudal middle frontal | -1.93E-03 | 1.86E-03 | -1.04 | 0.30 | 0.52 |
| MCI | DNAmPhenoAge | Left | Precentral | -1.94E-03 | 1.97E-03 | -0.98 | 0.33 | 0.54 |
| MCI | DNAmPhenoAge | Left | Banks of the superior temporal sulcus | -2.31E-03 | 2.36E-03 | -0.98 | 0.33 | 0.54 |
| MCI | DNAmPhenoAge | Left | Postcentral | -1.26E-03 | 1.55E-03 | -0.81 | 0.42 | 0.61 |
| MCI | DNAmPhenoAge | Left | Supramarginal | -1.53E-03 | 1.94E-03 | -0.79 | 0.43 | 0.61 |
| MCI | DNAmPhenoAge | Left | Isthmus cingulate | -1.90E-03 | 2.42E-03 | -0.78 | 0.43 | 0.61 |
| MCI | DNAmPhenoAge | Left | Superior parietal | -1.26E-03 | 1.72E-03 | -0.73 | 0.47 | 0.62 |
| MCI | DNAmPhenoAge | Left | Posterior Cingulate | -1.24E-03 | 1.87E-03 | -0.66 | 0.51 | 0.65 |
| MCI | DNAmPhenoAge | Left | Insula | -1.22E-03 | 2.23E-03 | -0.55 | 0.59 | 0.72 |
| MCI | DNAmPhenoAge | Left | Frontalpole | -1.32E-03 | 3.13E-03 | -0.42 | 0.67 | 0.81 |
| MCI | DNAmPhenoAge | Left | Caudal anterior cingulate | 1.36E-03 | 3.66E-03 | 0.37 | 0.71 | 0.82 |
| MCI | DNAmPhenoAge | Left | Lateral occipital | -3.87E-04 | 1.90E-03 | -0.20 | 0.84 | 0.93 |
| MCI | DNAmPhenoAge | Left | Fusiform | 1.60E-04 | 2.31E-03 | 0.07 | 0.94 | 0.97 |
| MCI | DNAmPhenoAge | Left | Paracentral | -1.47E-05 | 1.72E-03 | -0.01 | 0.99 | 0.99 |
| MCI | DNAmPhenoAge | Right | Isthmus cingulate | -5.68E-03 | 2.61E-03 | -2.17 | 0.03 | 0.33 |
| MCI | DNAmPhenoAge | Right | Superior temporal | -4.04E-03 | 1.90E-03 | -2.13 | 0.03 | 0.33 |
| MCI | DNAmPhenoAge | Right | Precuneus | -3.16E-03 | 1.61E-03 | -1.97 | 0.05 | 0.33 |
| MCI | DNAmPhenoAge | Right | Transverse temporal | -5.35E-03 | 2.88E-03 | -1.86 | 0.07 | 0.33 |
| MCI | DNAmPhenoAge | Right | Middle temporal | -3.43E-03 | 1.90E-03 | -1.80 | 0.07 | 0.33 |
| MCI | DNAmPhenoAge | Right | Lingual | -2.88E-03 | 1.68E-03 | -1.71 | 0.09 | 0.34 |
| MCI | DNAmPhenoAge | Right | Parahippocampal | -6.04E-03 | 3.69E-03 | -1.64 | 0.10 | 0.34 |
| MCI | DNAmPhenoAge | Right | Precentral | -3.03E-03 | 1.86E-03 | -1.63 | 0.11 | 0.34 |
| MCI | DNAmPhenoAge | Right | Lateral occipital | -3.20E-03 | 2.05E-03 | -1.56 | 0.12 | 0.36 |
| MCI | DNAmPhenoAge | Right | Pars orbitalis | -3.62E-03 | 2.38E-03 | -1.53 | 0.13 | 0.36 |
| MCI | DNAmPhenoAge | Right | Medial orbitofrontal | -2.94E-03 | 2.02E-03 | -1.45 | 0.15 | 0.39 |
| MCI | DNAmPhenoAge | Right | Banks of the superior temporal sulcus | -2.78E-03 | 1.98E-03 | -1.40 | 0.16 | 0.41 |
| MCI | DNAmPhenoAge | Right | Insula | -2.84E-03 | 2.12E-03 | -1.34 | 0.18 | 0.43 |
| MCI | DNAmPhenoAge | Right | Pars opercularis | -2.20E-03 | 1.66E-03 | -1.33 | 0.19 | 0.43 |
| MCI | DNAmPhenoAge | Right | Cuneus | -2.13E-03 | 1.69E-03 | -1.26 | 0.21 | 0.44 |
| MCI | DNAmPhenoAge | Right | Lateral orbitofrontal | -2.28E-03 | 1.84E-03 | -1.24 | 0.22 | 0.45 |
| MCI | DNAmPhenoAge | Right | Supramarginal | -2.00E-03 | 1.69E-03 | -1.19 | 0.24 | 0.47 |
| MCI | DNAmPhenoAge | Right | Pericalcarine | -1.61E-03 | 1.52E-03 | -1.06 | 0.29 | 0.52 |
| MCI | DNAmPhenoAge | Right | Temporal pole | -4.27E-03 | 4.75E-03 | -0.90 | 0.37 | 0.59 |
| MCI | DNAmPhenoAge | Right | Inferior parietal | -1.70E-03 | 1.91E-03 | -0.89 | 0.37 | 0.59 |
| MCI | DNAmPhenoAge | Right | Pars triangularis | -1.48E-03 | 1.70E-03 | -0.87 | 0.38 | 0.59 |
| MCI | DNAmPhenoAge | Right | Caudal middle frontal | -1.42E-03 | 1.80E-03 | -0.79 | 0.43 | 0.61 |
| MCI | DNAmPhenoAge | Right | Rostral middle frontal | -1.05E-03 | 1.37E-03 | -0.77 | 0.44 | 0.62 |
| MCI | DNAmPhenoAge | Right | Entorhinal | -4.40E-03 | 5.86E-03 | -0.75 | 0.45 | 0.62 |
| MCI | DNAmPhenoAge | Right | Postcentral | -1.06E-03 | 1.51E-03 | -0.70 | 0.48 | 0.63 |
| MCI | DNAmPhenoAge | Right | Superior frontal | -1.07E-03 | 1.64E-03 | -0.65 | 0.52 | 0.65 |
| MCI | DNAmPhenoAge | Right | Superior parietal | -9.12E-04 | 1.87E-03 | -0.49 | 0.63 | 0.76 |
| MCI | DNAmPhenoAge | Right | Inferior temporal | -7.74E-04 | 2.05E-03 | -0.38 | 0.71 | 0.82 |
| MCI | DNAmPhenoAge | Right | Fusiform | -7.78E-04 | 2.23E-03 | -0.35 | 0.73 | 0.82 |
| MCI | DNAmPhenoAge | Right | Posterior Cingulate | 3.57E-04 | 2.05E-03 | 0.17 | 0.86 | 0.93 |
| MCI | DNAmPhenoAge | Right | Frontalpole | -5.07E-04 | 3.13E-03 | -0.16 | 0.87 | 0.93 |
| MCI | DNAmPhenoAge | Right | Caudal anterior cingulate | 4.89E-04 | 3.13E-03 | 0.16 | 0.88 | 0.93 |
| MCI | DNAmPhenoAge | Right | Paracentral | -1.34E-04 | 1.83E-03 | -0.07 | 0.94 | 0.97 |
| MCI | DNAmPhenoAge | Right | Rostral anterior cingulate | -1.16E-04 | 2.85E-03 | -0.04 | 0.97 | 0.98 |
| AD | DNAmGrimAge | Left | Paracentral | 1.88E-02 | 1.08E-02 | 1.75 | 0.09 | 0.77 |
| AD | DNAmGrimAge | Left | Precuneus | 1.19E-02 | 7.64E-03 | 1.56 | 0.13 | 0.86 |
| AD | DNAmGrimAge | Left | Lateral occipital | 1.30E-02 | 9.05E-03 | 1.43 | 0.16 | 0.86 |
| AD | DNAmGrimAge | Left | Inferior parietal | 1.41E-02 | 1.01E-02 | 1.39 | 0.17 | 0.86 |
| AD | DNAmGrimAge | Left | Temporal pole | 4.13E-02 | 2.98E-02 | 1.39 | 0.18 | 0.86 |
| AD | DNAmGrimAge | Left | Pars triangularis | -1.51E-02 | 1.11E-02 | -1.36 | 0.18 | 0.86 |
| AD | DNAmGrimAge | Left | Entorhinal | 3.89E-02 | 2.87E-02 | 1.36 | 0.18 | 0.86 |
| AD | DNAmGrimAge | Left | Caudal anterior cingulate | 2.59E-02 | 2.05E-02 | 1.26 | 0.22 | 0.86 |
| AD | DNAmGrimAge | Left | Rostral anterior cingulate | 2.21E-02 | 1.81E-02 | 1.22 | 0.23 | 0.86 |
| AD | DNAmGrimAge | Left | Isthmus cingulate | 1.42E-02 | 1.23E-02 | 1.15 | 0.26 | 0.86 |
| AD | DNAmGrimAge | Left | Lingual | 6.28E-03 | 5.50E-03 | 1.14 | 0.26 | 0.86 |
| AD | DNAmGrimAge | Left | Banks of the superior temporal sulcus | 1.18E-02 | 1.04E-02 | 1.13 | 0.27 | 0.86 |
| AD | DNAmGrimAge | Left | Middle temporal | 1.34E-02 | 1.27E-02 | 1.06 | 0.30 | 0.92 |
| AD | DNAmGrimAge | Left | Posterior Cingulate | 8.83E-03 | 9.44E-03 | 0.94 | 0.36 | 0.96 |
| AD | DNAmGrimAge | Left | Superior parietal | 8.14E-03 | 9.11E-03 | 0.89 | 0.38 | 0.96 |
| AD | DNAmGrimAge | Left | Pars opercularis | -7.89E-03 | 9.26E-03 | -0.85 | 0.40 | 0.96 |
| AD | DNAmGrimAge | Left | Lateral orbitofrontal | -7.10E-03 | 9.98E-03 | -0.71 | 0.48 | 0.96 |
| AD | DNAmGrimAge | Left | Medial orbitofrontal | 6.36E-03 | 9.23E-03 | 0.69 | 0.50 | 0.96 |
| AD | DNAmGrimAge | Left | Superior frontal | 6.88E-03 | 1.06E-02 | 0.65 | 0.52 | 0.96 |
| AD | DNAmGrimAge | Left | Pericalcarine | -5.25E-03 | 8.16E-03 | -0.64 | 0.52 | 0.96 |
| AD | DNAmGrimAge | Left | Precentral | 6.37E-03 | 1.01E-02 | 0.63 | 0.53 | 0.96 |
| AD | DNAmGrimAge | Left | Supramarginal | 4.86E-03 | 8.70E-03 | 0.56 | 0.58 | 0.96 |
| AD | DNAmGrimAge | Left | Cuneus | 3.90E-03 | 7.80E-03 | 0.50 | 0.62 | 0.96 |
| AD | DNAmGrimAge | Left | Postcentral | 3.92E-03 | 7.86E-03 | 0.50 | 0.62 | 0.96 |
| AD | DNAmGrimAge | Left | Parahippocampal | -1.12E-02 | 2.34E-02 | -0.48 | 0.63 | 0.96 |
| AD | DNAmGrimAge | Left | Caudal middle frontal | 5.21E-03 | 1.09E-02 | 0.48 | 0.64 | 0.96 |
| AD | DNAmGrimAge | Left | Transverse temporal | 5.61E-03 | 1.18E-02 | 0.48 | 0.64 | 0.96 |
| AD | DNAmGrimAge | Left | Inferior temporal | 4.54E-03 | 1.27E-02 | 0.36 | 0.72 | 0.96 |
| AD | DNAmGrimAge | Left | Frontalpole | 5.85E-03 | 1.65E-02 | 0.35 | 0.73 | 0.96 |
| AD | DNAmGrimAge | Left | Fusiform | 4.54E-03 | 1.31E-02 | 0.35 | 0.73 | 0.96 |
| AD | DNAmGrimAge | Left | Pars orbitalis | -3.80E-03 | 1.31E-02 | -0.29 | 0.77 | 0.96 |
| AD | DNAmGrimAge | Left | Rostral middle frontal | -2.30E-03 | 8.07E-03 | -0.28 | 0.78 | 0.96 |
| AD | DNAmGrimAge | Left | Superior temporal | 2.30E-03 | 1.10E-02 | 0.21 | 0.84 | 0.96 |
| AD | DNAmGrimAge | Left | Insula | 9.08E-04 | 1.27E-02 | 0.07 | 0.94 | 0.97 |
| AD | DNAmGrimAge | Right | Pericalcarine | 1.58E-02 | 6.48E-03 | 2.44 | 0.02 | 0.58 |
| AD | DNAmGrimAge | Right | Paracentral | 2.25E-02 | 9.38E-03 | 2.40 | 0.02 | 0.58 |
| AD | DNAmGrimAge | Right | Cuneus | 1.71E-02 | 7.85E-03 | 2.18 | 0.04 | 0.58 |
| AD | DNAmGrimAge | Right | Lingual | 1.39E-02 | 6.51E-03 | 2.13 | 0.04 | 0.58 |
| AD | DNAmGrimAge | Right | Lateral occipital | 1.84E-02 | 8.68E-03 | 2.12 | 0.04 | 0.58 |
| AD | DNAmGrimAge | Right | Pars orbitalis | -2.05E-02 | 1.10E-02 | -1.86 | 0.07 | 0.77 |
| AD | DNAmGrimAge | Right | Transverse temporal | 2.24E-02 | 1.27E-02 | 1.76 | 0.09 | 0.77 |
| AD | DNAmGrimAge | Right | Precuneus | 9.90E-03 | 8.36E-03 | 1.18 | 0.25 | 0.86 |
| AD | DNAmGrimAge | Right | Inferior parietal | 1.17E-02 | 9.93E-03 | 1.17 | 0.25 | 0.86 |
| AD | DNAmGrimAge | Right | Superior parietal | 8.54E-03 | 8.57E-03 | 1.00 | 0.33 | 0.96 |
| AD | DNAmGrimAge | Right | Parahippocampal | 1.47E-02 | 1.81E-02 | 0.81 | 0.42 | 0.96 |
| AD | DNAmGrimAge | Right | Frontalpole | 1.22E-02 | 1.81E-02 | 0.67 | 0.51 | 0.96 |
| AD | DNAmGrimAge | Right | Superior frontal | -6.38E-03 | 9.69E-03 | -0.66 | 0.52 | 0.96 |
| AD | DNAmGrimAge | Right | Posterior Cingulate | -6.60E-03 | 1.07E-02 | -0.62 | 0.54 | 0.96 |
| AD | DNAmGrimAge | Right | Lateral orbitofrontal | -5.94E-03 | 1.03E-02 | -0.57 | 0.57 | 0.96 |
| AD | DNAmGrimAge | Right | Middle temporal | 6.28E-03 | 1.39E-02 | 0.45 | 0.66 | 0.96 |
| AD | DNAmGrimAge | Right | Precentral | 4.56E-03 | 1.02E-02 | 0.45 | 0.66 | 0.96 |
| AD | DNAmGrimAge | Right | Supramarginal | 3.51E-03 | 8.10E-03 | 0.43 | 0.67 | 0.96 |
| AD | DNAmGrimAge | Right | Superior temporal | 3.61E-03 | 1.01E-02 | 0.36 | 0.72 | 0.96 |
| AD | DNAmGrimAge | Right | Rostral anterior cingulate | -5.19E-03 | 1.48E-02 | -0.35 | 0.73 | 0.96 |
| AD | DNAmGrimAge | Right | Pars opercularis | -2.26E-03 | 7.95E-03 | -0.28 | 0.78 | 0.96 |
| AD | DNAmGrimAge | Right | Entorhinal | 1.05E-02 | 3.78E-02 | 0.28 | 0.78 | 0.96 |
| AD | DNAmGrimAge | Right | Caudal anterior cingulate | -4.41E-03 | 1.63E-02 | -0.27 | 0.79 | 0.96 |
| AD | DNAmGrimAge | Right | Pars triangularis | 2.70E-03 | 1.02E-02 | 0.26 | 0.79 | 0.96 |
| AD | DNAmGrimAge | Right | Postcentral | 1.93E-03 | 8.30E-03 | 0.23 | 0.82 | 0.96 |
| AD | DNAmGrimAge | Right | Fusiform | 2.82E-03 | 1.36E-02 | 0.21 | 0.84 | 0.96 |
| AD | DNAmGrimAge | Right | Rostral middle frontal | -1.63E-03 | 8.58E-03 | -0.19 | 0.85 | 0.96 |
| AD | DNAmGrimAge | Right | Banks of the superior temporal sulcus | 1.82E-03 | 1.00E-02 | 0.18 | 0.86 | 0.96 |
| AD | DNAmGrimAge | Right | Caudal middle frontal | 1.57E-03 | 1.07E-02 | 0.15 | 0.88 | 0.97 |
| AD | DNAmGrimAge | Right | Insula | -1.47E-03 | 1.44E-02 | -0.10 | 0.92 | 0.97 |
| AD | DNAmGrimAge | Right | Medial orbitofrontal | -1.04E-03 | 1.09E-02 | -0.10 | 0.92 | 0.97 |
| AD | DNAmGrimAge | Right | Inferior temporal | -1.10E-03 | 1.48E-02 | -0.07 | 0.94 | 0.97 |
| AD | DNAmGrimAge | Right | Temporal pole | 7.14E-04 | 3.57E-02 | 0.02 | 0.98 | 1.00 |
| AD | DNAmGrimAge | Right | Isthmus cingulate | 2.20E-05 | 1.05E-02 | 0.00 | 1.00 | 1.00 |
| ALL | DNAmGrimAge | Left | Medial orbitofrontal | -8.24E-03 | 2.63E-03 | -3.13 | 1.90E-03 | 0.06 |
| ALL | DNAmGrimAge | Left | Rostral middle frontal | -6.09E-03 | 2.15E-03 | -2.84 | 4.84E-03 | 0.08 |
| ALL | DNAmGrimAge | Left | Caudal middle frontal | -7.10E-03 | 2.66E-03 | -2.66 | 0.01 | 0.08 |
| ALL | DNAmGrimAge | Left | Precentral | -7.57E-03 | 2.87E-03 | -2.64 | 0.01 | 0.08 |
| ALL | DNAmGrimAge | Left | Superior temporal | -8.27E-03 | 3.20E-03 | -2.58 | 0.01 | 0.08 |
| ALL | DNAmGrimAge | Left | Pars triangularis | -6.74E-03 | 2.77E-03 | -2.43 | 0.02 | 0.08 |
| ALL | DNAmGrimAge | Left | Middle temporal | -7.84E-03 | 3.23E-03 | -2.43 | 0.02 | 0.08 |
| ALL | DNAmGrimAge | Left | Superior frontal | -6.00E-03 | 2.57E-03 | -2.34 | 0.02 | 0.10 |
| ALL | DNAmGrimAge | Left | Lateral orbitofrontal | -6.01E-03 | 2.61E-03 | -2.30 | 0.02 | 0.10 |
| ALL | DNAmGrimAge | Left | Pars opercularis | -4.40E-03 | 2.36E-03 | -1.86 | 0.06 | 0.24 |
| ALL | DNAmGrimAge | Left | Postcentral | -3.98E-03 | 2.32E-03 | -1.71 | 0.09 | 0.27 |
| ALL | DNAmGrimAge | Left | Superior parietal | -4.24E-03 | 2.48E-03 | -1.71 | 0.09 | 0.27 |
| ALL | DNAmGrimAge | Left | Insula | -5.35E-03 | 3.19E-03 | -1.68 | 0.09 | 0.27 |
| ALL | DNAmGrimAge | Left | Supramarginal | -4.49E-03 | 2.73E-03 | -1.64 | 0.10 | 0.28 |
| ALL | DNAmGrimAge | Left | Banks of the superior temporal sulcus | -5.36E-03 | 3.37E-03 | -1.59 | 0.11 | 0.29 |
| ALL | DNAmGrimAge | Left | Inferior parietal | -4.01E-03 | 2.72E-03 | -1.47 | 0.14 | 0.32 |
| ALL | DNAmGrimAge | Left | Transverse temporal | -5.55E-03 | 3.88E-03 | -1.43 | 0.15 | 0.32 |
| ALL | DNAmGrimAge | Left | Isthmus cingulate | -4.83E-03 | 3.55E-03 | -1.36 | 0.17 | 0.34 |
| ALL | DNAmGrimAge | Left | Fusiform | -4.25E-03 | 3.20E-03 | -1.33 | 0.18 | 0.35 |
| ALL | DNAmGrimAge | Left | Posterior Cingulate | -3.41E-03 | 2.80E-03 | -1.22 | 0.22 | 0.41 |
| ALL | DNAmGrimAge | Left | Frontalpole | -5.29E-03 | 4.66E-03 | -1.14 | 0.26 | 0.45 |
| ALL | DNAmGrimAge | Left | Pars orbitalis | -3.38E-03 | 3.60E-03 | -0.94 | 0.35 | 0.54 |
| ALL | DNAmGrimAge | Left | Entorhinal | -6.86E-03 | 7.71E-03 | -0.89 | 0.37 | 0.55 |
| ALL | DNAmGrimAge | Left | Precuneus | -1.92E-03 | 2.41E-03 | -0.79 | 0.43 | 0.61 |
| ALL | DNAmGrimAge | Left | Rostral anterior cingulate | -2.92E-03 | 4.52E-03 | -0.64 | 0.52 | 0.72 |
| ALL | DNAmGrimAge | Left | Lingual | -1.30E-03 | 2.26E-03 | -0.57 | 0.57 | 0.75 |
| ALL | DNAmGrimAge | Left | Temporal pole | -3.68E-03 | 6.46E-03 | -0.57 | 0.57 | 0.75 |
| ALL | DNAmGrimAge | Left | Inferior temporal | -1.87E-03 | 3.31E-03 | -0.57 | 0.57 | 0.75 |
| ALL | DNAmGrimAge | Left | Pericalcarine | -1.14E-03 | 2.27E-03 | -0.50 | 0.62 | 0.77 |
| ALL | DNAmGrimAge | Left | Cuneus | -1.19E-03 | 2.38E-03 | -0.50 | 0.62 | 0.77 |
| ALL | DNAmGrimAge | Left | Paracentral | -1.16E-03 | 2.73E-03 | -0.43 | 0.67 | 0.80 |
| ALL | DNAmGrimAge | Left | Lateral occipital | -5.27E-04 | 2.63E-03 | -0.20 | 0.84 | 0.93 |
| ALL | DNAmGrimAge | Left | Parahippocampal | -1.88E-04 | 6.24E-03 | -0.03 | 0.98 | 0.99 |
| ALL | DNAmGrimAge | Left | Caudal anterior cingulate | -1.60E-04 | 5.55E-03 | -0.03 | 0.98 | 0.99 |
| ALL | DNAmGrimAge | Right | Pars orbitalis | -1.19E-02 | 3.50E-03 | -3.40 | 7.67E-04 | 0.05 |
| ALL | DNAmGrimAge | Right | Superior frontal | -6.48E-03 | 2.39E-03 | -2.71 | 0.01 | 0.08 |
| ALL | DNAmGrimAge | Right | Medial orbitofrontal | -8.24E-03 | 3.04E-03 | -2.71 | 0.01 | 0.08 |
| ALL | DNAmGrimAge | Right | Precentral | -6.70E-03 | 2.73E-03 | -2.45 | 0.01 | 0.08 |
| ALL | DNAmGrimAge | Right | Rostral middle frontal | -5.20E-03 | 2.13E-03 | -2.45 | 0.01 | 0.08 |
| ALL | DNAmGrimAge | Right | Lateral orbitofrontal | -6.65E-03 | 2.75E-03 | -2.42 | 0.02 | 0.08 |
| ALL | DNAmGrimAge | Right | Isthmus cingulate | -8.13E-03 | 3.68E-03 | -2.21 | 0.03 | 0.12 |
| ALL | DNAmGrimAge | Right | Pars opercularis | -5.13E-03 | 2.44E-03 | -2.11 | 0.04 | 0.14 |
| ALL | DNAmGrimAge | Right | Supramarginal | -4.49E-03 | 2.51E-03 | -1.79 | 0.07 | 0.27 |
| ALL | DNAmGrimAge | Right | Insula | -5.66E-03 | 3.24E-03 | -1.74 | 0.08 | 0.27 |
| ALL | DNAmGrimAge | Right | Rostral anterior cingulate | -7.68E-03 | 4.56E-03 | -1.68 | 0.09 | 0.27 |
| ALL | DNAmGrimAge | Right | Middle temporal | -4.70E-03 | 2.97E-03 | -1.58 | 0.11 | 0.29 |
| ALL | DNAmGrimAge | Right | Caudal middle frontal | -4.06E-03 | 2.60E-03 | -1.56 | 0.12 | 0.29 |
| ALL | DNAmGrimAge | Right | Inferior parietal | -4.24E-03 | 2.77E-03 | -1.53 | 0.13 | 0.30 |
| ALL | DNAmGrimAge | Right | Pars triangularis | -3.70E-03 | 2.58E-03 | -1.43 | 0.15 | 0.32 |
| ALL | DNAmGrimAge | Right | Postcentral | -3.30E-03 | 2.32E-03 | -1.42 | 0.16 | 0.32 |
| ALL | DNAmGrimAge | Right | Caudal anterior cingulate | -6.75E-03 | 4.90E-03 | -1.38 | 0.17 | 0.34 |
| ALL | DNAmGrimAge | Right | Superior temporal | -3.25E-03 | 2.72E-03 | -1.19 | 0.23 | 0.42 |
| ALL | DNAmGrimAge | Right | Precuneus | -2.50E-03 | 2.45E-03 | -1.02 | 0.31 | 0.52 |
| ALL | DNAmGrimAge | Right | Banks of the superior temporal sulcus | -2.88E-03 | 2.86E-03 | -1.01 | 0.31 | 0.52 |
| ALL | DNAmGrimAge | Right | Frontalpole | -4.63E-03 | 4.64E-03 | -1.00 | 0.32 | 0.52 |
| ALL | DNAmGrimAge | Right | Superior parietal | -2.56E-03 | 2.63E-03 | -0.97 | 0.33 | 0.52 |
| ALL | DNAmGrimAge | Right | Paracentral | -2.62E-03 | 2.85E-03 | -0.92 | 0.36 | 0.54 |
| ALL | DNAmGrimAge | Right | Fusiform | -2.48E-03 | 3.10E-03 | -0.80 | 0.42 | 0.61 |
| ALL | DNAmGrimAge | Right | Parahippocampal | -2.56E-03 | 5.14E-03 | -0.50 | 0.62 | 0.77 |
| ALL | DNAmGrimAge | Right | Transverse temporal | -1.79E-03 | 4.21E-03 | -0.42 | 0.67 | 0.80 |
| ALL | DNAmGrimAge | Right | Lingual | -8.76E-04 | 2.38E-03 | -0.37 | 0.71 | 0.84 |
| ALL | DNAmGrimAge | Right | Lateral occipital | 8.18E-04 | 2.78E-03 | 0.29 | 0.77 | 0.89 |
| ALL | DNAmGrimAge | Right | Temporal pole | -1.63E-03 | 7.19E-03 | -0.23 | 0.82 | 0.93 |
| ALL | DNAmGrimAge | Right | Entorhinal | -1.67E-03 | 8.44E-03 | -0.20 | 0.84 | 0.93 |
| ALL | DNAmGrimAge | Right | Inferior temporal | -4.38E-04 | 3.17E-03 | -0.14 | 0.89 | 0.96 |
| ALL | DNAmGrimAge | Right | Posterior Cingulate | -3.77E-04 | 2.99E-03 | -0.13 | 0.90 | 0.96 |
| ALL | DNAmGrimAge | Right | Pericalcarine | -7.38E-05 | 2.20E-03 | -0.03 | 0.97 | 0.99 |
| ALL | DNAmGrimAge | Right | Cuneus | 4.34E-05 | 2.51E-03 | 0.02 | 0.99 | 0.99 |
| CN | DNAmGrimAge | Left | Caudal middle frontal | -1.46E-02 | 4.04E-03 | -3.60 | 5.09E-04 | 0.01 |
| CN | DNAmGrimAge | Left | Rostral middle frontal | -1.35E-02 | 3.76E-03 | -3.58 | 5.42E-04 | 0.01 |
| CN | DNAmGrimAge | Left | Superior parietal | -1.25E-02 | 3.64E-03 | -3.43 | 8.88E-04 | 0.01 |
| CN | DNAmGrimAge | Left | Superior frontal | -1.34E-02 | 4.07E-03 | -3.29 | 1.43E-03 | 0.01 |
| CN | DNAmGrimAge | Left | Precentral | -1.48E-02 | 4.77E-03 | -3.09 | 2.60E-03 | 0.01 |
| CN | DNAmGrimAge | Left | Medial orbitofrontal | -1.48E-02 | 4.81E-03 | -3.09 | 2.66E-03 | 0.01 |
| CN | DNAmGrimAge | Left | Supramarginal | -1.26E-02 | 4.17E-03 | -3.03 | 3.16E-03 | 0.01 |
| CN | DNAmGrimAge | Left | Lateral orbitofrontal | -1.33E-02 | 4.48E-03 | -2.98 | 3.70E-03 | 0.02 |
| CN | DNAmGrimAge | Left | Paracentral | -1.36E-02 | 4.76E-03 | -2.86 | 0.01 | 0.02 |
| CN | DNAmGrimAge | Left | Postcentral | -1.13E-02 | 3.96E-03 | -2.85 | 0.01 | 0.02 |
| CN | DNAmGrimAge | Left | Cuneus | -1.16E-02 | 4.27E-03 | -2.71 | 0.01 | 0.02 |
| CN | DNAmGrimAge | Left | Precuneus | -9.91E-03 | 3.81E-03 | -2.60 | 0.01 | 0.03 |
| CN | DNAmGrimAge | Left | Superior temporal | -1.24E-02 | 4.98E-03 | -2.49 | 0.01 | 0.04 |
| CN | DNAmGrimAge | Left | Lateral occipital | -9.20E-03 | 3.90E-03 | -2.36 | 0.02 | 0.05 |
| CN | DNAmGrimAge | Left | Pars triangularis | -1.26E-02 | 5.38E-03 | -2.33 | 0.02 | 0.05 |
| CN | DNAmGrimAge | Left | Inferior parietal | -1.05E-02 | 4.51E-03 | -2.32 | 0.02 | 0.05 |
| CN | DNAmGrimAge | Left | Middle temporal | -1.08E-02 | 4.69E-03 | -2.31 | 0.02 | 0.05 |
| CN | DNAmGrimAge | Left | Posterior Cingulate | -1.12E-02 | 5.01E-03 | -2.24 | 0.03 | 0.05 |
| CN | DNAmGrimAge | Left | Fusiform | -9.01E-03 | 4.16E-03 | -2.17 | 0.03 | 0.05 |
| CN | DNAmGrimAge | Left | Lingual | -6.64E-03 | 3.84E-03 | -1.73 | 0.09 | 0.12 |
| CN | DNAmGrimAge | Left | Frontalpole | -1.33E-02 | 7.80E-03 | -1.71 | 0.09 | 0.12 |
| CN | DNAmGrimAge | Left | Pars opercularis | -7.01E-03 | 4.22E-03 | -1.66 | 0.10 | 0.13 |
| CN | DNAmGrimAge | Left | Banks of the superior temporal sulcus | -8.62E-03 | 5.59E-03 | -1.54 | 0.13 | 0.16 |
| CN | DNAmGrimAge | Left | Isthmus cingulate | -8.06E-03 | 5.64E-03 | -1.43 | 0.16 | 0.20 |
| CN | DNAmGrimAge | Left | Insula | -6.92E-03 | 5.00E-03 | -1.38 | 0.17 | 0.21 |
| CN | DNAmGrimAge | Left | Pars orbitalis | -6.25E-03 | 6.66E-03 | -0.94 | 0.35 | 0.42 |
| CN | DNAmGrimAge | Left | Transverse temporal | -5.61E-03 | 6.64E-03 | -0.84 | 0.40 | 0.45 |
| CN | DNAmGrimAge | Left | Rostral anterior cingulate | -6.67E-03 | 7.91E-03 | -0.84 | 0.40 | 0.45 |
| CN | DNAmGrimAge | Left | Pericalcarine | -3.46E-03 | 4.11E-03 | -0.84 | 0.40 | 0.45 |
| CN | DNAmGrimAge | Left | Caudal anterior cingulate | -6.62E-03 | 9.91E-03 | -0.67 | 0.51 | 0.55 |
| CN | DNAmGrimAge | Left | Inferior temporal | -2.83E-03 | 4.68E-03 | -0.61 | 0.55 | 0.58 |
| CN | DNAmGrimAge | Left | Entorhinal | -5.76E-03 | 9.65E-03 | -0.60 | 0.55 | 0.58 |
| CN | DNAmGrimAge | Left | Parahippocampal | 4.77E-03 | 9.99E-03 | 0.48 | 0.63 | 0.64 |
| CN | DNAmGrimAge | Left | Temporal pole | -3.37E-03 | 8.33E-03 | -0.40 | 0.69 | 0.69 |
| CN | DNAmGrimAge | Right | Precentral | -1.64E-02 | 4.49E-03 | -3.65 | 4.32E-04 | 0.01 |
| CN | DNAmGrimAge | Right | Superior parietal | -1.41E-02 | 3.94E-03 | -3.59 | 5.37E-04 | 0.01 |
| CN | DNAmGrimAge | Right | Superior frontal | -1.32E-02 | 3.79E-03 | -3.49 | 7.43E-04 | 0.01 |
| CN | DNAmGrimAge | Right | Lateral orbitofrontal | -1.53E-02 | 4.75E-03 | -3.23 | 1.71E-03 | 0.01 |
| CN | DNAmGrimAge | Right | Middle temporal | -1.46E-02 | 4.51E-03 | -3.22 | 1.74E-03 | 0.01 |
| CN | DNAmGrimAge | Right | Rostral middle frontal | -1.21E-02 | 3.81E-03 | -3.18 | 1.97E-03 | 0.01 |
| CN | DNAmGrimAge | Right | Caudal middle frontal | -1.25E-02 | 4.08E-03 | -3.07 | 2.78E-03 | 0.01 |
| CN | DNAmGrimAge | Right | Precuneus | -1.28E-02 | 4.17E-03 | -3.06 | 2.89E-03 | 0.01 |
| CN | DNAmGrimAge | Right | Inferior parietal | -1.24E-02 | 4.37E-03 | -2.85 | 0.01 | 0.02 |
| CN | DNAmGrimAge | Right | Inferior temporal | -1.32E-02 | 4.80E-03 | -2.75 | 0.01 | 0.02 |
| CN | DNAmGrimAge | Right | Postcentral | -1.12E-02 | 4.10E-03 | -2.74 | 0.01 | 0.02 |
| CN | DNAmGrimAge | Right | Frontalpole | -1.97E-02 | 7.53E-03 | -2.62 | 0.01 | 0.03 |
| CN | DNAmGrimAge | Right | Rostral anterior cingulate | -2.24E-02 | 8.74E-03 | -2.56 | 0.01 | 0.03 |
| CN | DNAmGrimAge | Right | Paracentral | -1.31E-02 | 5.27E-03 | -2.48 | 0.01 | 0.04 |
| CN | DNAmGrimAge | Right | Isthmus cingulate | -1.49E-02 | 6.18E-03 | -2.41 | 0.02 | 0.04 |
| CN | DNAmGrimAge | Right | Medial orbitofrontal | -1.31E-02 | 5.45E-03 | -2.40 | 0.02 | 0.04 |
| CN | DNAmGrimAge | Right | Supramarginal | -1.02E-02 | 4.40E-03 | -2.31 | 0.02 | 0.05 |
| CN | DNAmGrimAge | Right | Cuneus | -1.01E-02 | 4.43E-03 | -2.29 | 0.02 | 0.05 |
| CN | DNAmGrimAge | Right | Superior temporal | -8.79E-03 | 3.92E-03 | -2.24 | 0.03 | 0.05 |
| CN | DNAmGrimAge | Right | Pars opercularis | -9.55E-03 | 4.27E-03 | -2.24 | 0.03 | 0.05 |
| CN | DNAmGrimAge | Right | Pars triangularis | -1.02E-02 | 4.60E-03 | -2.23 | 0.03 | 0.05 |
| CN | DNAmGrimAge | Right | Fusiform | -9.09E-03 | 4.16E-03 | -2.19 | 0.03 | 0.05 |
| CN | DNAmGrimAge | Right | Pars orbitalis | -1.39E-02 | 6.41E-03 | -2.16 | 0.03 | 0.05 |
| CN | DNAmGrimAge | Right | Pericalcarine | -7.67E-03 | 3.79E-03 | -2.02 | 0.05 | 0.07 |
| CN | DNAmGrimAge | Right | Banks of the superior temporal sulcus | -9.31E-03 | 4.78E-03 | -1.95 | 0.05 | 0.08 |
| CN | DNAmGrimAge | Right | Lingual | -7.43E-03 | 3.87E-03 | -1.92 | 0.06 | 0.09 |
| CN | DNAmGrimAge | Right | Caudal anterior cingulate | -1.79E-02 | 9.57E-03 | -1.87 | 0.06 | 0.10 |
| CN | DNAmGrimAge | Right | Lateral occipital | -7.13E-03 | 3.93E-03 | -1.81 | 0.07 | 0.11 |
| CN | DNAmGrimAge | Right | Transverse temporal | -1.28E-02 | 7.41E-03 | -1.73 | 0.09 | 0.12 |
| CN | DNAmGrimAge | Right | Insula | -8.20E-03 | 5.36E-03 | -1.53 | 0.13 | 0.17 |
| CN | DNAmGrimAge | Right | Posterior Cingulate | -4.97E-03 | 5.25E-03 | -0.95 | 0.35 | 0.42 |
| CN | DNAmGrimAge | Right | Entorhinal | -9.76E-03 | 1.16E-02 | -0.84 | 0.40 | 0.45 |
| CN | DNAmGrimAge | Right | Temporal pole | -6.74E-03 | 9.90E-03 | -0.68 | 0.50 | 0.55 |
| CN | DNAmGrimAge | Right | Parahippocampal | -3.88E-03 | 7.60E-03 | -0.51 | 0.61 | 0.63 |
| MCI | DNAmGrimAge | Left | Medial orbitofrontal | -6.74E-03 | 3.33E-03 | -2.02 | 0.04 | 0.92 |
| MCI | DNAmGrimAge | Left | Middle temporal | -8.68E-03 | 4.48E-03 | -1.94 | 0.05 | 0.92 |
| MCI | DNAmGrimAge | Left | Superior temporal | -7.08E-03 | 4.55E-03 | -1.56 | 0.12 | 1.00 |
| MCI | DNAmGrimAge | Left | Precentral | -5.70E-03 | 3.88E-03 | -1.47 | 0.14 | 1.00 |
| MCI | DNAmGrimAge | Left | Caudal middle frontal | -4.76E-03 | 3.67E-03 | -1.30 | 0.20 | 1.00 |
| MCI | DNAmGrimAge | Left | Transverse temporal | -6.90E-03 | 5.34E-03 | -1.29 | 0.20 | 1.00 |
| MCI | DNAmGrimAge | Left | Entorhinal | -1.38E-02 | 1.08E-02 | -1.28 | 0.20 | 1.00 |
| MCI | DNAmGrimAge | Left | Isthmus cingulate | -6.01E-03 | 4.76E-03 | -1.26 | 0.21 | 1.00 |
| MCI | DNAmGrimAge | Left | Insula | -5.32E-03 | 4.39E-03 | -1.21 | 0.23 | 1.00 |
| MCI | DNAmGrimAge | Left | Banks of the superior temporal sulcus | -5.47E-03 | 4.65E-03 | -1.18 | 0.24 | 1.00 |
| MCI | DNAmGrimAge | Left | Rostral anterior cingulate | -6.51E-03 | 5.85E-03 | -1.11 | 0.27 | 1.00 |
| MCI | DNAmGrimAge | Left | Rostral middle frontal | -2.98E-03 | 2.82E-03 | -1.06 | 0.29 | 1.00 |
| MCI | DNAmGrimAge | Left | Superior frontal | -3.38E-03 | 3.45E-03 | -0.98 | 0.33 | 1.00 |
| MCI | DNAmGrimAge | Left | Cuneus | 3.06E-03 | 3.14E-03 | 0.97 | 0.33 | 1.00 |
| MCI | DNAmGrimAge | Left | Temporal pole | -8.38E-03 | 8.70E-03 | -0.96 | 0.34 | 1.00 |
| MCI | DNAmGrimAge | Left | Inferior parietal | -3.31E-03 | 3.61E-03 | -0.92 | 0.36 | 1.00 |
| MCI | DNAmGrimAge | Left | Pars opercularis | -2.81E-03 | 3.07E-03 | -0.92 | 0.36 | 1.00 |
| MCI | DNAmGrimAge | Left | Pars triangularis | -2.81E-03 | 3.44E-03 | -0.82 | 0.42 | 1.00 |
| MCI | DNAmGrimAge | Left | Lateral orbitofrontal | -2.38E-03 | 3.49E-03 | -0.68 | 0.50 | 1.00 |
| MCI | DNAmGrimAge | Left | Superior parietal | -2.08E-03 | 3.40E-03 | -0.61 | 0.54 | 1.00 |
| MCI | DNAmGrimAge | Left | Postcentral | -1.84E-03 | 3.06E-03 | -0.60 | 0.55 | 1.00 |
| MCI | DNAmGrimAge | Left | Posterior Cingulate | -2.14E-03 | 3.70E-03 | -0.58 | 0.56 | 1.00 |
| MCI | DNAmGrimAge | Left | Pars orbitalis | -2.37E-03 | 4.66E-03 | -0.51 | 0.61 | 1.00 |
| MCI | DNAmGrimAge | Left | Fusiform | -2.14E-03 | 4.55E-03 | -0.47 | 0.64 | 1.00 |
| MCI | DNAmGrimAge | Left | Lateral occipital | 1.72E-03 | 3.75E-03 | 0.46 | 0.65 | 1.00 |
| MCI | DNAmGrimAge | Left | Paracentral | 1.51E-03 | 3.40E-03 | 0.45 | 0.66 | 1.00 |
| MCI | DNAmGrimAge | Left | Supramarginal | -1.56E-03 | 3.83E-03 | -0.41 | 0.68 | 1.00 |
| MCI | DNAmGrimAge | Left | Inferior temporal | -1.44E-03 | 4.69E-03 | -0.31 | 0.76 | 1.00 |
| MCI | DNAmGrimAge | Left | Caudal anterior cingulate | -2.18E-03 | 7.23E-03 | -0.30 | 0.76 | 1.00 |
| MCI | DNAmGrimAge | Left | Precuneus | -7.66E-04 | 3.30E-03 | -0.23 | 0.82 | 1.00 |
| MCI | DNAmGrimAge | Left | Lingual | 7.07E-04 | 3.23E-03 | 0.22 | 0.83 | 1.00 |
| MCI | DNAmGrimAge | Left | Frontalpole | -1.17E-03 | 6.19E-03 | -0.19 | 0.85 | 1.00 |
| MCI | DNAmGrimAge | Left | Parahippocampal | -1.35E-03 | 8.62E-03 | -0.16 | 0.88 | 1.00 |
| MCI | DNAmGrimAge | Left | Pericalcarine | 7.91E-05 | 3.04E-03 | 0.03 | 0.98 | 1.00 |
| MCI | DNAmGrimAge | Right | Pars orbitalis | -1.13E-02 | 4.64E-03 | -2.43 | 0.02 | 0.92 |
| MCI | DNAmGrimAge | Right | Inferior temporal | 8.47E-03 | 3.99E-03 | 2.12 | 0.04 | 0.92 |
| MCI | DNAmGrimAge | Right | Medial orbitofrontal | -7.27E-03 | 3.98E-03 | -1.83 | 0.07 | 0.94 |
| MCI | DNAmGrimAge | Right | Pars opercularis | -4.67E-03 | 3.27E-03 | -1.43 | 0.15 | 1.00 |
| MCI | DNAmGrimAge | Right | Cuneus | 3.20E-03 | 3.33E-03 | 0.96 | 0.34 | 1.00 |
| MCI | DNAmGrimAge | Right | Isthmus cingulate | -4.91E-03 | 5.20E-03 | -0.94 | 0.35 | 1.00 |
| MCI | DNAmGrimAge | Right | Superior frontal | -2.88E-03 | 3.23E-03 | -0.89 | 0.37 | 1.00 |
| MCI | DNAmGrimAge | Right | Precentral | -3.09E-03 | 3.69E-03 | -0.84 | 0.40 | 1.00 |
| MCI | DNAmGrimAge | Right | Insula | -3.36E-03 | 4.19E-03 | -0.80 | 0.42 | 1.00 |
| MCI | DNAmGrimAge | Right | Lateral occipital | 3.08E-03 | 4.06E-03 | 0.76 | 0.45 | 1.00 |
| MCI | DNAmGrimAge | Right | Posterior Cingulate | 3.03E-03 | 4.03E-03 | 0.75 | 0.45 | 1.00 |
| MCI | DNAmGrimAge | Right | Parahippocampal | -5.10E-03 | 7.33E-03 | -0.70 | 0.49 | 1.00 |
| MCI | DNAmGrimAge | Right | Rostral middle frontal | -1.70E-03 | 2.70E-03 | -0.63 | 0.53 | 1.00 |
| MCI | DNAmGrimAge | Right | Supramarginal | -1.79E-03 | 3.34E-03 | -0.54 | 0.59 | 1.00 |
| MCI | DNAmGrimAge | Right | Lateral orbitofrontal | -1.83E-03 | 3.64E-03 | -0.50 | 0.62 | 1.00 |
| MCI | DNAmGrimAge | Right | Superior parietal | 1.77E-03 | 3.69E-03 | 0.48 | 0.63 | 1.00 |
| MCI | DNAmGrimAge | Right | Temporal pole | 4.27E-03 | 9.40E-03 | 0.45 | 0.65 | 1.00 |
| MCI | DNAmGrimAge | Right | Pars triangularis | -1.46E-03 | 3.36E-03 | -0.44 | 0.66 | 1.00 |
| MCI | DNAmGrimAge | Right | Inferior parietal | -1.58E-03 | 3.77E-03 | -0.42 | 0.68 | 1.00 |
| MCI | DNAmGrimAge | Right | Pericalcarine | 1.20E-03 | 3.01E-03 | 0.40 | 0.69 | 1.00 |
| MCI | DNAmGrimAge | Right | Rostral anterior cingulate | -1.92E-03 | 5.63E-03 | -0.34 | 0.73 | 1.00 |
| MCI | DNAmGrimAge | Right | Transverse temporal | 1.81E-03 | 5.74E-03 | 0.32 | 0.75 | 1.00 |
| MCI | DNAmGrimAge | Right | Fusiform | 1.22E-03 | 4.39E-03 | 0.28 | 0.78 | 1.00 |
| MCI | DNAmGrimAge | Right | Caudal anterior cingulate | -1.36E-03 | 6.18E-03 | -0.22 | 0.83 | 1.00 |
| MCI | DNAmGrimAge | Right | Caudal middle frontal | 7.12E-04 | 3.56E-03 | 0.20 | 0.84 | 1.00 |
| MCI | DNAmGrimAge | Right | Postcentral | -5.27E-04 | 2.98E-03 | -0.18 | 0.86 | 1.00 |
| MCI | DNAmGrimAge | Right | Precuneus | 4.49E-04 | 3.20E-03 | 0.14 | 0.89 | 1.00 |
| MCI | DNAmGrimAge | Right | Entorhinal | 1.08E-03 | 1.16E-02 | 0.09 | 0.93 | 1.00 |
| MCI | DNAmGrimAge | Right | Banks of the superior temporal sulcus | 3.26E-04 | 3.94E-03 | 0.08 | 0.93 | 1.00 |
| MCI | DNAmGrimAge | Right | Middle temporal | 2.68E-04 | 3.79E-03 | 0.07 | 0.94 | 1.00 |
| MCI | DNAmGrimAge | Right | Frontalpole | 1.93E-04 | 6.18E-03 | 0.03 | 0.98 | 1.00 |
| MCI | DNAmGrimAge | Right | Superior temporal | -1.02E-04 | 3.79E-03 | -0.03 | 0.98 | 1.00 |
| MCI | DNAmGrimAge | Right | Paracentral | -4.16E-05 | 3.61E-03 | -0.01 | 0.99 | 1.00 |
| MCI | DNAmGrimAge | Right | Lingual | 1.93E-06 | 3.35E-03 | 0.00 | 1.00 | 1.00 |

**Table S6 Legend:** Associations between DNAmPhenoAge and DNAmGrimAge with cortical thickness stratified by diagnosis (i.e., CN, MCI, and AD) as well as the overall cohort (“ALL) are shown. All analyses were performed using multiple regression covarying for chronologic age, sex, education, CDR-SB score, and APOE ε4 dose.
